# RNA activation of *CEBPA* improves leukemia treatment

**DOI:** 10.1016/j.omtn.2025.102643

**Published:** 2025-08-20

**Authors:** Olivia Kovecses, Bahram Sharif-Askari, Cristobal Gonzalez-Losada, Vikash Reebye, Bríd M. Ryan, Albert Kwok, Nagy A. Habib, Nathan W. Luedtke, François E. Mercier, Maureen McKeague

## Main text

(Molecular Therapy: Nucleic Acids *36*, 102611; September 2025)

In the originally published article, Drs. Nagy Habib and Albert Kwok were inadvertently omitted from the author list. Following discussions with all co-authors and their collaborators at MiNA Therapeutics, the authors reviewed their contributions and confirmed that they meet the journal’s authorship criteria. All parties are in agreement to add them to the author list. The authors apologize for any inconvenience. Drs. Nagy Habib and Albert Kwok contributed significantly to project design. Their contributions included facilitating the provision of study materials, providing scientific context for experimental results, and offering perspective on the findings in relation to clinical context. The authors apologize for this omission.

