# Supplementary material for: RNA activation of CEBPA improves leukemia treatment
Source: Mol Ther Nucleic Acids. 2025 Jun 16;36(3):102611. doi: 10.1016/j.omtn.2025.102611 (PMC12271619; doi:10.1016/j.omtn.2025.102611)
Supplement: Document S1. Figures S1–S9 and Table S1 [file mmc1.pdf]

OMTN, Volume *36*

Supplemental information

RNA activation of *CEBPA*

improves leukemia treatment

Olivia Kovecses, Bahram Sharif-Askari, Cristobal Gonzalez-Losada, Vikash Reebye, Bríd M. Ryan, Nathan W. Luedtke, François E. Mercier, and Maureen McKeague

SUPPLEMENTAL METHODS

Cy3-MTL-CEBPA dose-uptake assay

Cells were seeded at a concentration of 5 x 10^5 cells/mL 24 hours prior to start of treatment. Cells were washed once in PBS and resuspend in serum-free RPMI-1640 with 1% ABAM. Cy3-MTL-CEBPA was added to culture media for a final concentration of 0 µg/mL, 0.025 µg/mL, 0.050 µg/mL, 0.01 µg/mL, 0.2 µg/mL, 0.4 µg/mL, 0.8 µg/mL, 1.6 µg/mL, 3.125 µg/mL, 6.25 µg/mL, 12.5 µg/mL, or 25 µg/mL. Cells were left in Cy3-MTL-CEBPA-containing media overnight (~16 hours). Cells were then collected, washed twice with PBS, and stained for live and dead cells with the LIVE/DEAD fixable green dead cell stain kit (Thermo Fisher Scientific) according to the manufacturer's instructions. Flow cytometry analysis was then performed on samples using the Sony ID7000 Full spectrum flow cytometer. Live cells were gated, and the median fluorescence intensity (MFI) of Cy3 was obtained.

Cy3-MTL-CEBPA uptake over time

Cell viability was determined with Trypan Blue staining (Gibco) and cells were seeded at a concentration of 5x10^5 cells/mL 16-24 hours prior to start of treatment. Cells were washed once in PBS and resuspend in serum-free RPMI-1640 with 1% ABAM. Cy3-MTL-CEBPA was added to culture media for a final concentration of 2 µg/mL and cells were incubated for 10 minutes, 30 minutes, 1 hour, 2 hours, 4 hours, 6 hours, or 24 hours. Cells were then collected, washed twice with PBS, and stained for live and dead cells with the LIVE/DEAD fixable green dead cell stain kit (Thermo Fisher Scientific) according to the manufacturer's instructions. Flow cytometry analysis was then performed on samples using the Sony ID7000 Full spectrum flow cytometer.

SUPPLEMENTAL FIGURES

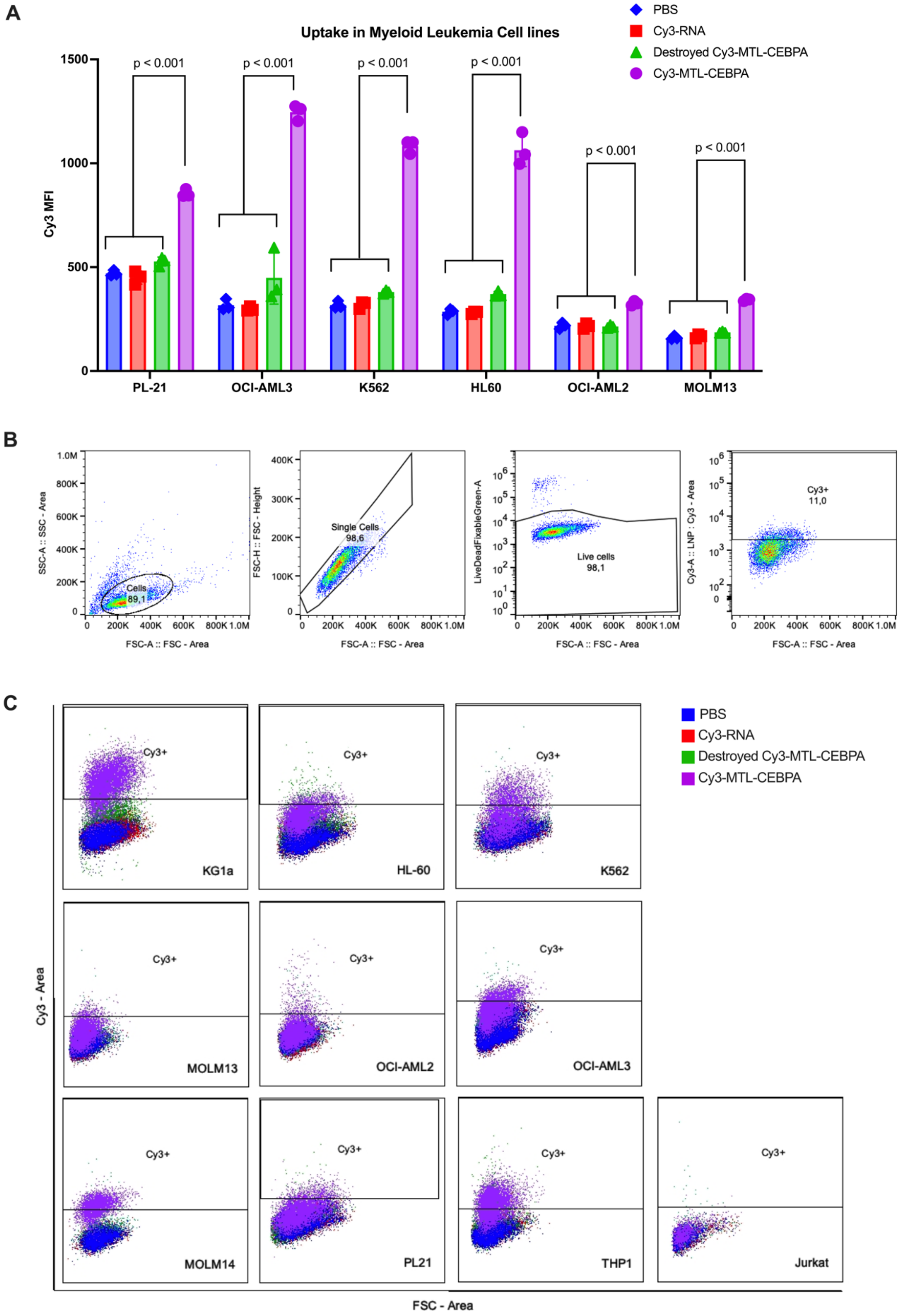

Figure S1. Uptake of Cy3-MTL-CEBPA in AML cell lines. (A) Delivery of fluorescently tagged CEBPA-51 saRNA in myeloid leukemia cell lines. Two-way ANOVA with Holm-Sidak Multiple Comparison Test; n=3. Data represented as mean ± SD. **(B)** Representative gating strategy for quantification of % of Cy3+ cells. **(C)** Representative dot plot maps of cell lines tested.

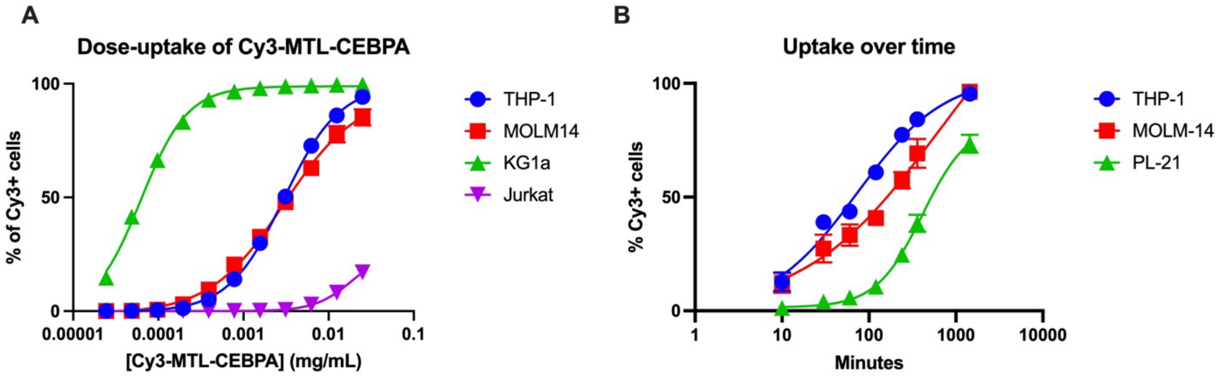

Figure S2. Cy3-MTL-CEBPA kinetics in AML cell lines. (A) Dose-response relationship of Cy3-MTL-CEBPA in THP-1, MOLM14, KG1a, and Jurkat cell lines. Data represented as mean ± SD. **(B)** Uptake of 2 μg/mL Cy3-MTL-CEBPA over time in THP-1, MOLM14, and PL21 cell lines. Data represented as mean ± SD.

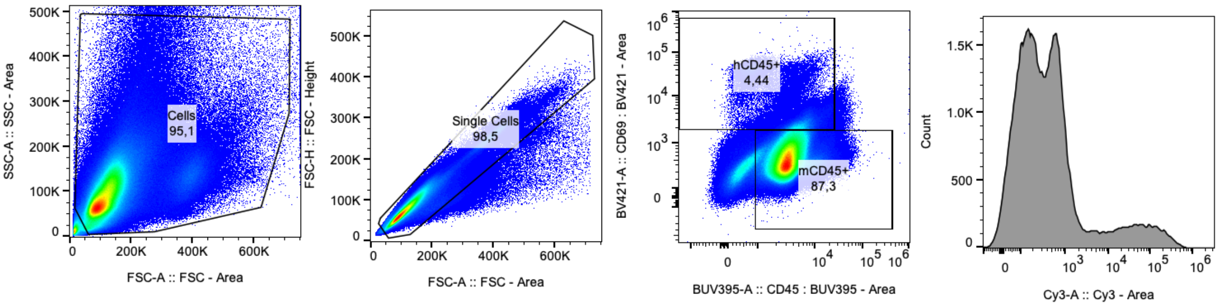

Figure S3. ***In vivo*** **uptake of Cy3-MTL-CEBPA in PDX mouse model.** Gating Strategy for quantification of Cy3 median fluorescence intensity (MFI) for Cy3-MTL-CEBPA uptake in PDX models.

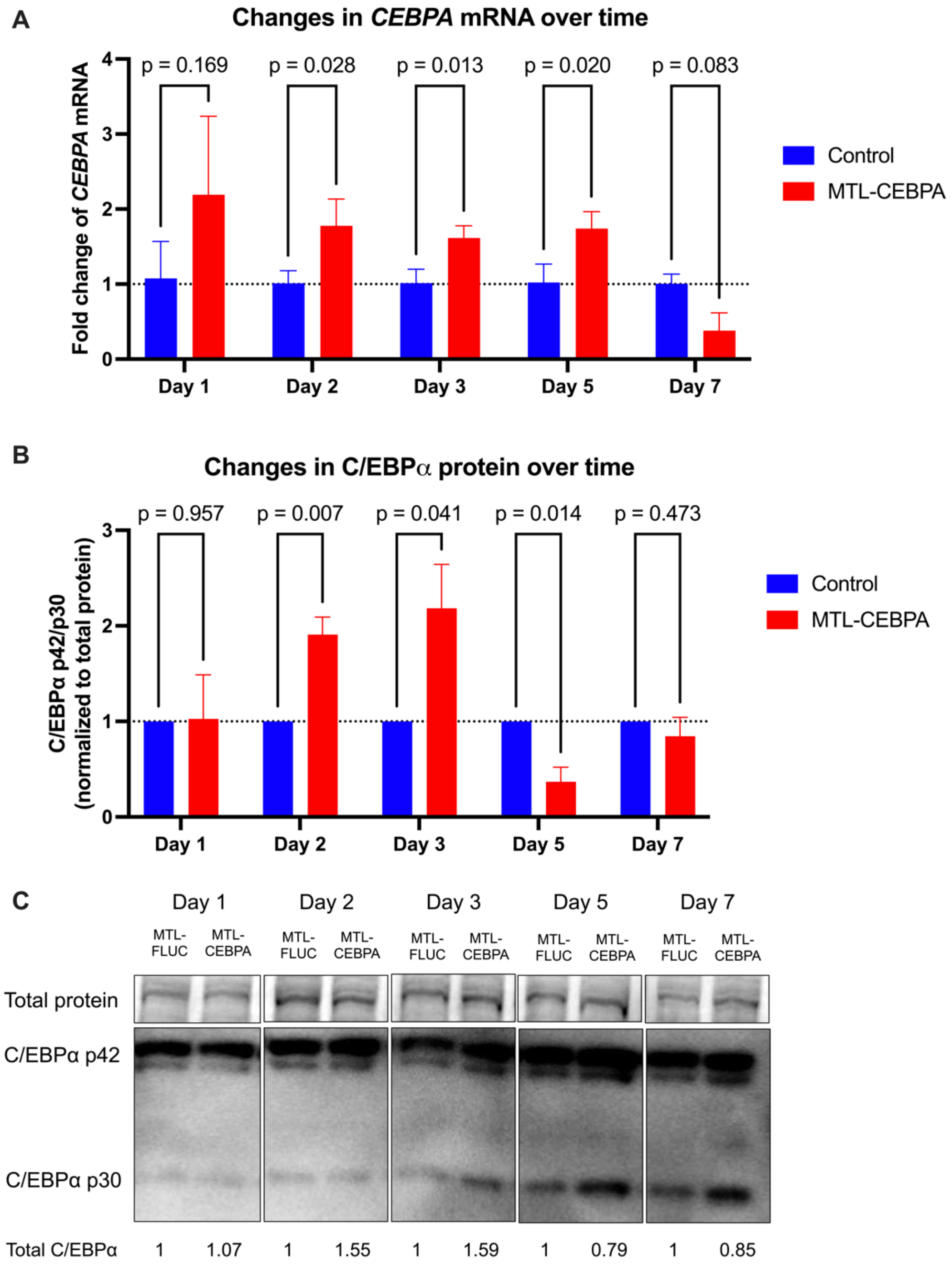

Figure S4. Changes in *CEBPA* expression over time. (A) *CEBPA* mRNA expression in MOLM-14 cells over time after a single treatment with 10 µg/mL MTL-CEBPA. Two-tailed unpaired student t-test; n=3. Data represented as mean ± SEM. **(B)** C/EBPα p42/p30 isoform ratio in MOLM-14 cells over time after a single treatment with 10 µg/mL MTL-CEBPA. Two-tailed unpaired student t-test; n=3. Data represented as mean ± SEM. **(C)** Representative western blot of C/EBPα over time. Relative total C/EBPα values written below blot.

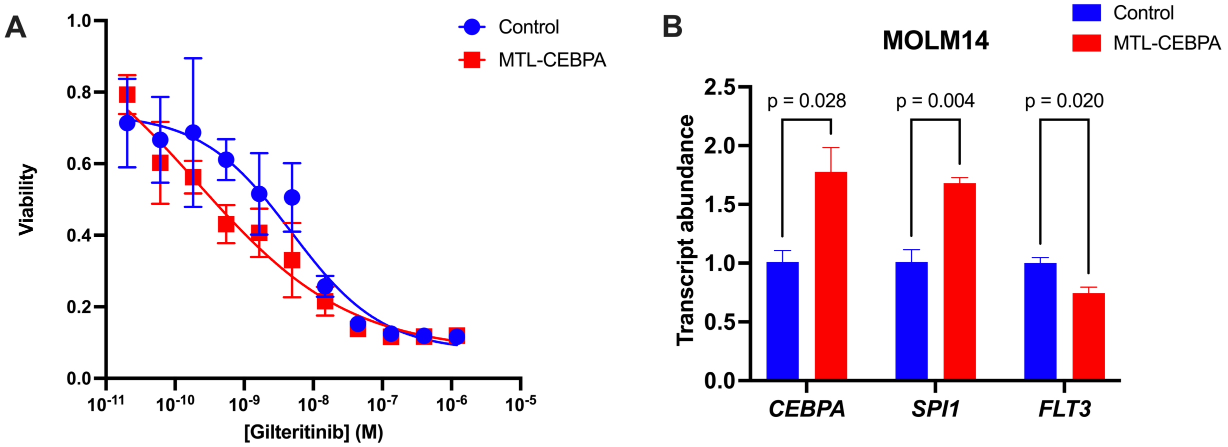

Figure S5. Therapeutic effect of MTL-CEBPA in MOLM14. (A) Gilteritinib dose-response relationship of MOLM14-venus cells treated with 2 µg/mL of MTL-CEBPA (blue) vs cells treated with equal concentration of control (MTL-FLUC). Data represented as mean ± SD. **(B)** Change in *CEBPA, SPI1,* and *FLT3* mRNA expression in MOLM-14 cells 72 hours after 10 µg/mL MTL-CEPBA treatment as compared to control (MTL-FLUC). Two-tailed unpaired student t-test; n=3. Data represented as mean ± SEM.

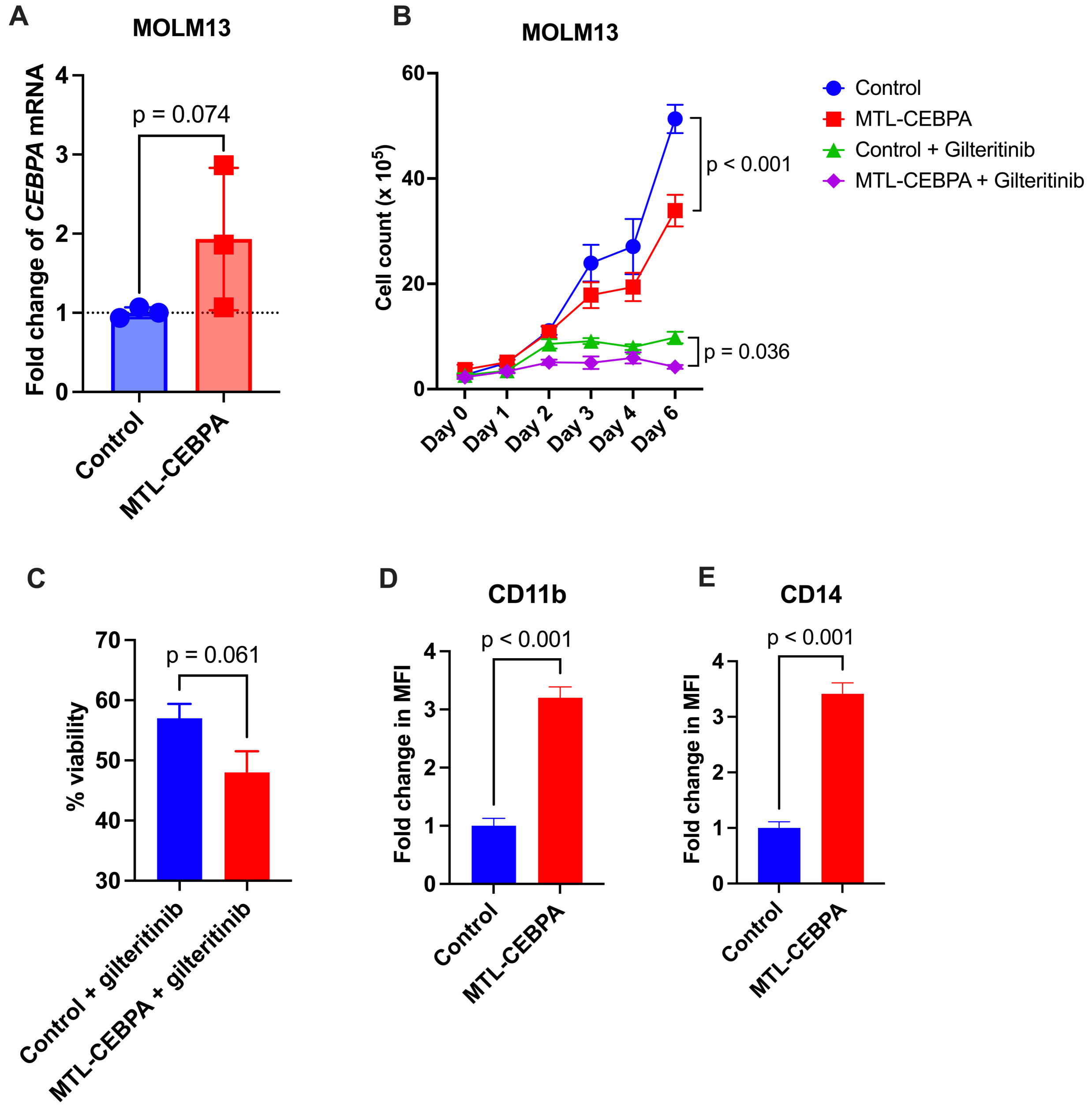

Figure S6. Therapeutic effect of MTL-CEBPA in MOLM-13. (A) MTL-CEBPA upregulates *CEBPA* mRNA, relative to cells treated with equal concentration (2 μg/mL) of control (MTL-FLUC) in MOLM13. Two-way unpaired student t-test; n=3. Data represented as mean ± SD. **(B)** Growth curves for MOLM-13 cells following treatment with 10 μg/mL of MTL-CEBPA or control (MTL-FLUC) with and without 5 nM gilteritinib. Two-way ANOVA with Holm-Sidak Multiple Comparison Test; n=6. Data represented as mean ± SEM. **(C)** Percent viability of cells on day 6. Two-way unpaired student t-test; n=6. Data represented as mean ± SEM. **(D)** Fold change in CD11b MFI and **(E)** CD14 MFI in MOLM-13 cells following treatment with 10 μg/mL MTL-CEBPA or control (untreated). Two-way unpaired student t-test; n=2-3. Data represented as mean ± SEM.

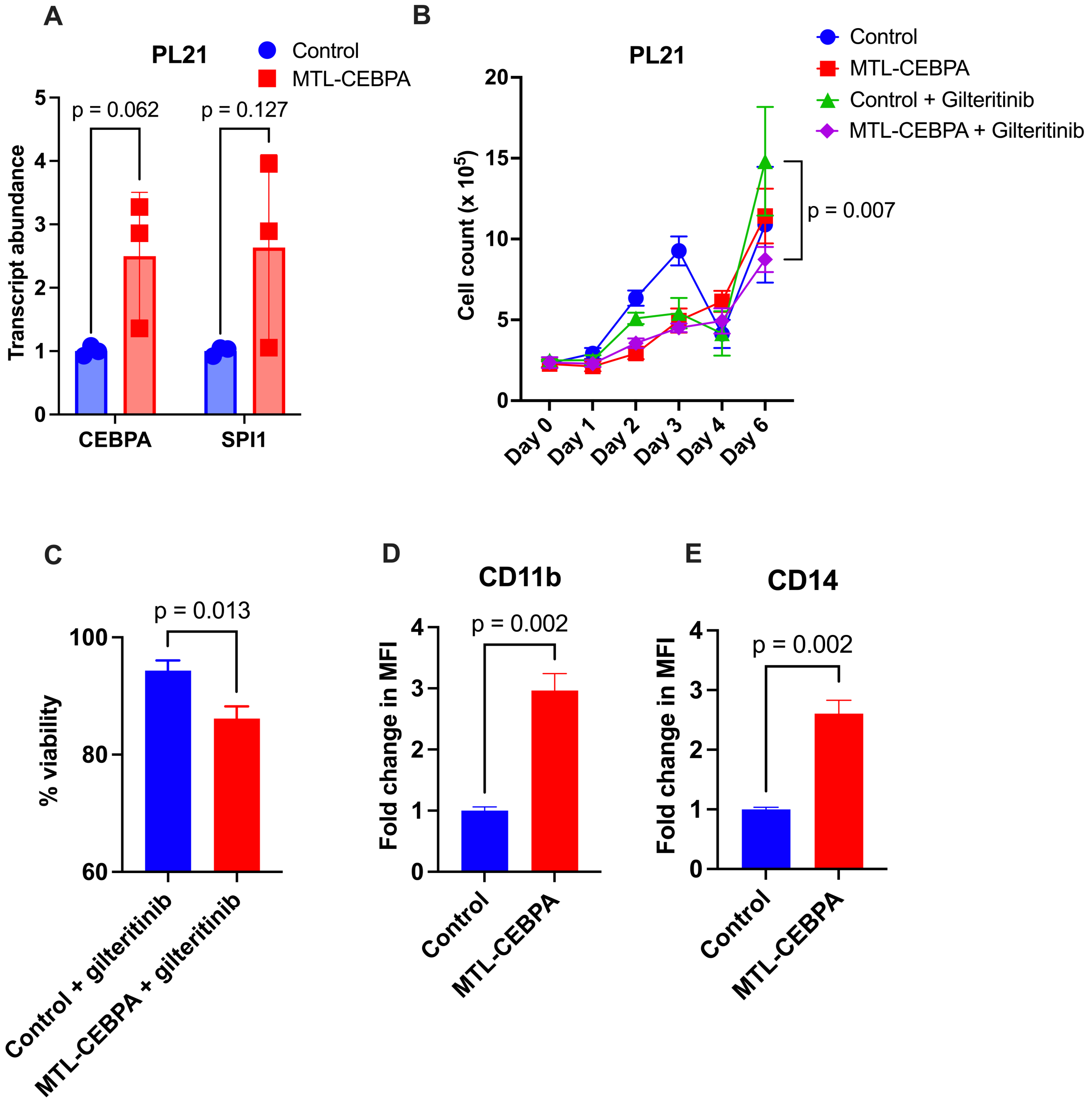

Figure S7. Therapeutic effect of MTL-CEBPA in PL-21. (A) MTL-CEBPA upregulates *CEBPA* and *SPI1* mRNA, relative to cells treated with equal concentration (2 µg/mL) of control (MTL-FLUC) in PL-21. Two-way unpaired student t-test; n=3. Data represented as mean ± SD. **(B)** Growth curves for PL-21 cells following treatment with 10 µg/mL of MTL-CEBPA or control (MTL-FLUC) with and without 5 nM gilteritinib. Two-way ANOVA with Holm-Sidak Multiple Comparison Test; n=6. Data represented as mean ± SEM. **(C)** Percent viability of cells on day 6. Two-way unpaired student t-test; n=6. Data represented as mean ± SEM. **(D)** Fold change in CD11b MFI and **(E)** CD14 MFI in PL-21 cells following treatment with 10 µg/mL MTL-CEBPA or control (untreated). Two-way unpaired student t-test; n=2-3. Data represented as mean ± SEM.

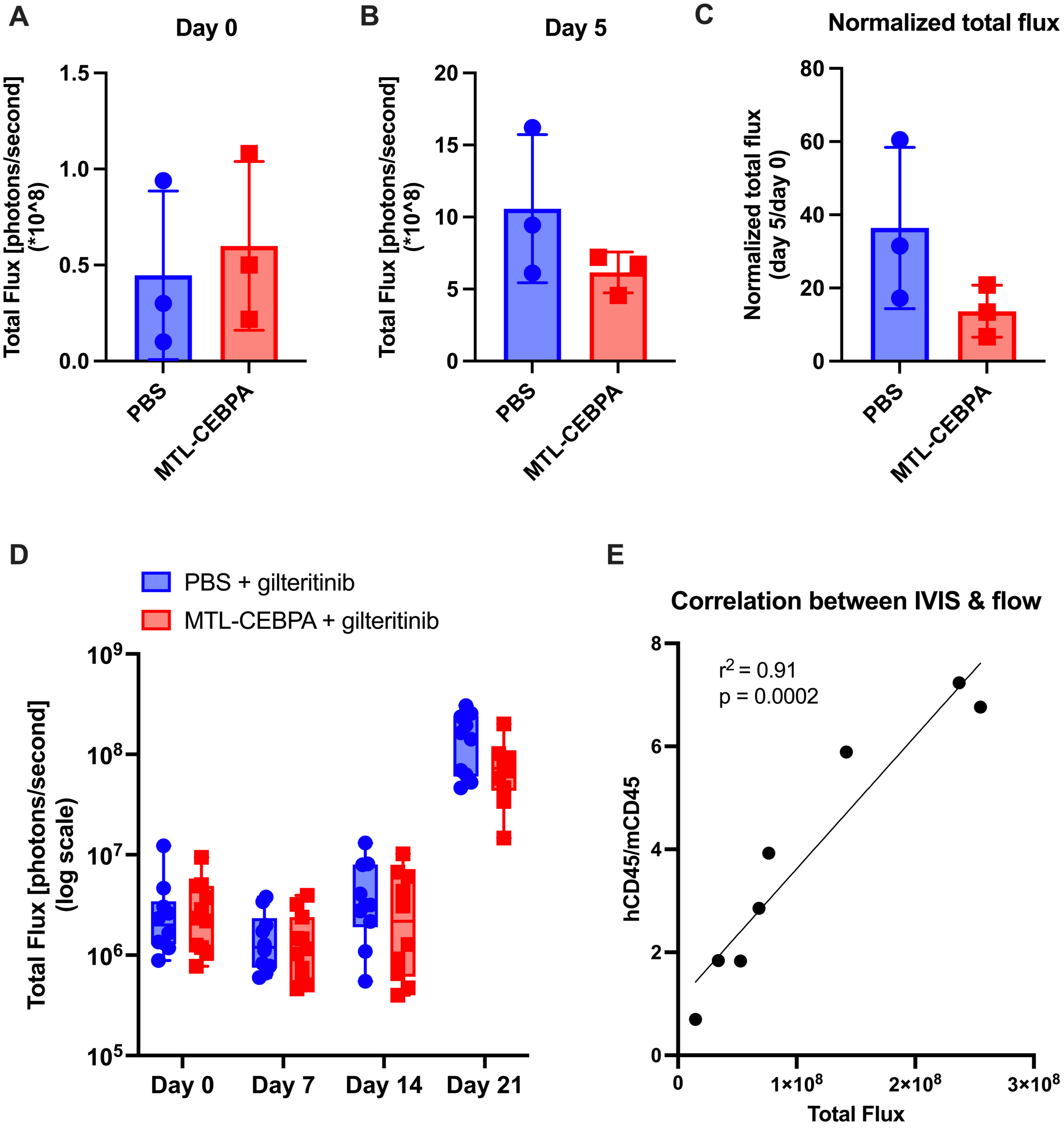

Figure S8. (A) Bioluminescence signal (total flux) measured on day 0 of MOLM-14-luc-engrafted NSG mice. **(B)** Total flux measured on day 5 of MOLM-14-luc-engrafted mice following intravenous administration of 3 mg/kg MTL-CEBPA or PBS every 2 days. **(C)** Normalized total flux for MOLM-14-luc-engrafted NSG mice. **(D)** Total flux values for results shown in Figure 5. **(E)** Correlation between total flux (obtained through in vivo imaging system (IVIS)) and ratio of human CD45 to mouse CD45 cells in the bone marrow of MOLM-14-luc-engrafted mice.

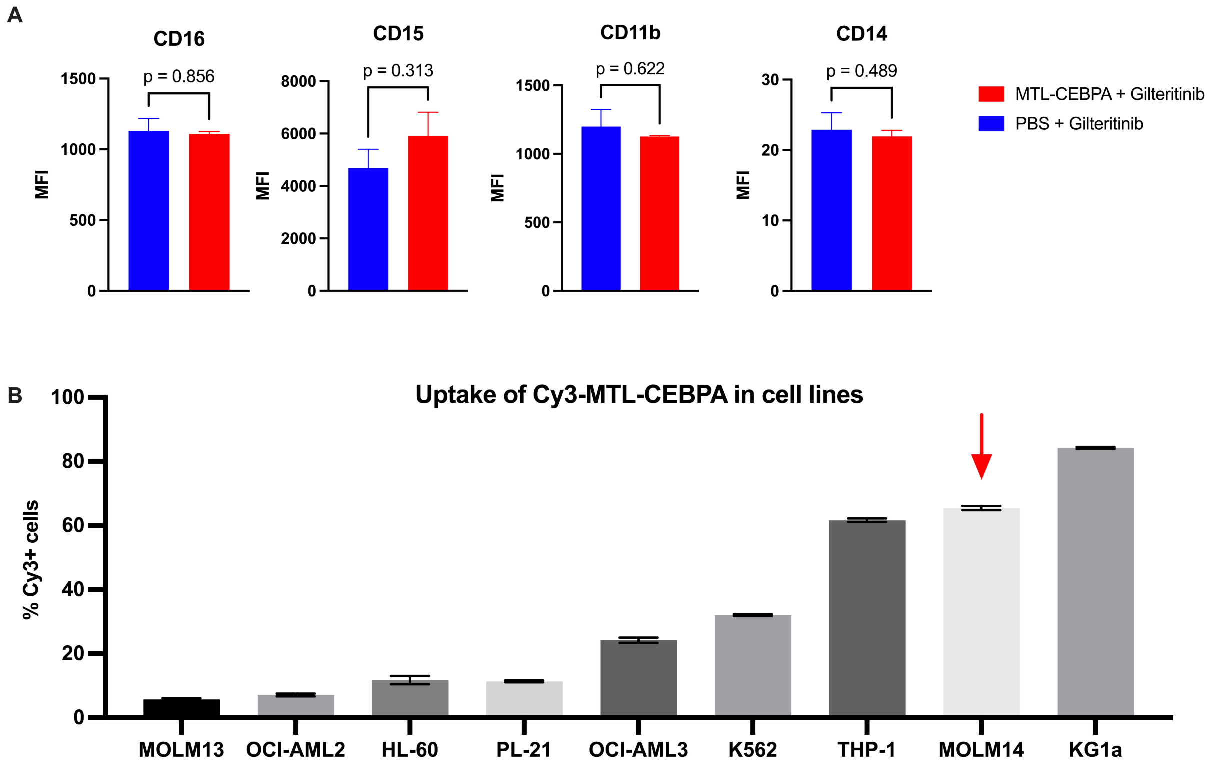

Figure S9. (A) Expression of myeloid-specific cell surface markers CD16, CD15, CD11b, CD14 in human MOLM-14-Ven-Luc cells isolated from the bone marrow of xenograft mice at day 21. **(B)** Percent uptake of Cy3-MTL-CEBPA in AML cell lines. Red arrow showing percent uptake for MOLM14 cell line.

SUPPLEMENTAL TABLES

Table S1. Summary table of FLT3 inhibitors used in study.

FLT3 inhibitor	Targets	Use in AML
gilteritinib	FLT3, AXL, ALK, and LTK[1]	Relapsed/refractor AML (ADMIRAL trial)[2]
midostaurin	FLT3, PKC, KDR, KIT, VEGFR, PDGFR[3]	Newly diagnosed AML (RATIFY trial)[4]
sorafenib	FLT3, RAF, RET, KIT, VEGFR, PDGFR[5]	Post-transplant maintenance (SORMAIN trial)[6]
quizartinib	FLT3, KIT, PDCFR[7]	Newly diagnosed AML (QuANTUM-First trial)[8]
ponatinib	FLT3, BCR-ABL, KIT, FGFR1, PDGFR[9]	Under evaluation (used in CML and Ph+ ALL)[10]
sunitinib	FLT3, PDGFR, VEGFR, KIT[11]	Under evaluation (currently used in solid tumours)
crenolanib	FLT3, PDGFR[12]	Under evaluation
lestaurtinib	FLT3, TRK, JAK2, PKC, KDR, PDGFR	Withdrawn from use[13]

SUPPLEMENTAL REFERENCES

1 Lee, L. Y., Hernandez, D., Rajkhowa, T., Smith, S. C., Raman, J. R., Nguyen, B., Small, D. & Levis, M. Preclinical studies of gilteritinib, a next-generation FLT3 inhibitor. *Blood* **129**, 257-260 (2017). https://doi.org/10.1182/blood-2016-10-745133

2 Perl, A. E., Martinelli, G., Cortes, J. E., Neubauer, A., Berman, E., Paolini, S., Montesinos, P., Baer, M. R., Larson, R. A., Ustun, C. *et al.* Gilteritinib or Chemotherapy for Relapsed or Refractory <i>FLT3</i>-Mutated AML. *New England Journal of Medicine* **381**, 1728-1740 (2019). https://doi.org/doi:10.1056/NEJMoa1902688

3 Schlenk, R. F. & Kayser, S. Midostaurin: A Multiple Tyrosine Kinases Inhibitor in Acute Myeloid Leukemia and Systemic Mastocytosis. *Recent Results Cancer Res* **212**, 199-214 (2018). https://doi.org/10.1007/978-3-319-91439-8_10

4 Stone, R. M., Mandrekar, S. J., Sanford, B. L., Laumann, K., Geyer, S., Bloomfield, C. D., Thiede, C., Prior, T. W., Döhner, K., Marcucci, G. *et al.* Midostaurin plus Chemotherapy for Acute Myeloid Leukemia with a <i>FLT3</i> Mutation. *New England Journal of Medicine* **377**, 454-464 (2017). https://doi.org/doi:10.1056/NEJMoa1614359

5 Zhu, Y.-j., Zheng, B., Wang, H.-y. & Chen, L. New knowledge of the mechanisms of sorafenib resistance in liver cancer. *Acta Pharmacologica Sinica* **38**, 614-622 (2017). https://doi.org/10.1038/aps.2017.5

6 Burchert, A., Bug, G., Fritz, L. V., Finke, J., Stelljes, M., Röllig, C., Wollmer, E., Wäsch, R., Bornhäuser, M., Berg, T. *et al.* Sorafenib Maintenance After Allogeneic Hematopoietic Stem Cell Transplantation for Acute Myeloid Leukemia With <i>FLT3</i>–Internal Tandem Duplication Mutation (SORMAIN). *Journal of Clinical Oncology* **38**, 2993-3002 (2020). https://doi.org/10.1200/jco.19.03345

7 Kampa-Schittenhelm, K. M., Heinrich, M. C., Akmut, F., Döhner, H., Döhner, K. & Schittenhelm, M. M. Quizartinib (AC220) is a potent second generation class III tyrosine kinase inhibitor that displays a distinct inhibition profile against mutant-FLT3, -PDGFRA and -KIT isoforms. *Molecular Cancer* **12**, 19 (2013). https://doi.org/10.1186/1476-4598-12-19

8 Erba, H. P., Montesinos, P., Kim, H.-J., Patkowska, E., Vrhovac, R., Žák, P., Wang, P.-N., Mitov, T., Hanyok, J., Kamel, Y. M. *et al.* Quizartinib plus chemotherapy in newly diagnosed patients with FLT3-internal-tandem-duplication-positive acute myeloid leukaemia (QuANTUM-First): a randomised, double-blind, placebo-controlled, phase 3 trial. *The Lancet* **401**, 1571-1583 (2023). https://doi.org/10.1016/S0140-6736(23)00464-6

9 Gao, Y., Ding, Y., Tai, X.-r., Zhang, C. & Wang, D. Ponatinib: An update on its drug targets, therapeutic potential and safety. *Biochimica et Biophysica Acta (BBA) - Reviews on Cancer* **1878**, 188949 (2023). https://doi.org/https://doi.org/10.1016/j.bbcan.2023.188949

10 Chevallier, P., Thiebaut, A., François, S., Chantepie, S., Rubio, M. T., Labussiere-Wallet, H., Brissot, E., Maillard, N., Huynh, A., Coiteux, V. *et al.* A phase II study of ponatinib for prevention of relapse after allotransplantation in FLT3 internal tandem duplication mutation positive (FLT3-ITD+) acute myeloid leukemia: the

PONALLO trial. *Haematologica* (2025). https://doi.org/10.3324/haematol.2025.287681
11 Yu, J., Jiang, P. Y. Z., Sun, H., Zhang, X., Jiang, Z., Li, Y. & Song, Y. Advances in targeted therapy for acute myeloid leukemia. *Biomarker Research* **8**, 17 (2020). https://doi.org/10.1186/s40364-020-00196-2
12 Galanis, A., Ma, H., Rajkhowa, T., Ramachandran, A., Small, D., Cortes, J. & Levis, M. Crenolanib is a potent inhibitor of FLT3 with activity against resistance-conferring point mutants. *Blood* **123**, 94-100 (2014). https://doi.org/10.1182/blood-2013-10-529313
13 Levis, M., Ravandi, F., Wang, E. S., Baer, M. R., Perl, A., Coutre, S., Erba, H., Stuart, R. K., Baccarani, M. & Cripe, L. D. Results from a randomized trial of salvage chemotherapy followed by lestaurtinib for FLT3 mutant AML patients in first relapse. *Blood* **114**, 788 (2009).
