# Supplementary material for: RNA activation of CEBPA improves leukemia treatment
Source: Mol Ther Nucleic Acids. 2025 Jun 16;36(3):102611. doi: 10.1016/j.omtn.2025.102611 (PMC12271619; doi:10.1016/j.omtn.2025.102611)
Supplement: Document S2. Article plus supplemental information [file mmc2.pdf]

Molecular Therapy
Nucleic Acids
Original Article

RNA activation of *CEBPA* improves leukemia treatment

Olivia Kovecses,[1] Bahram Sharif-Askari,[2] Cristobal Gonzalez-Losada,[3] Vikash Reebye,[4] Bríd M. Ryan,[4] Nathan W. Luedtke,[1,5] François E. Mercier,[2,3,6] and Maureen McKeague[1,5]

[1]Department of Pharmacology & Therapeutics, McGill University, Montreal, Québec H3G 1Y6, Canada; [2]Lady Davis Institute for Medical Research & Cancer Segal Center, Jewish General Hospital, Montreal, Québec H3T 1E1, Canada; [3]Division of Clinical and Translational Research, Department of Medicine, McGill University, Montreal, Québec H4A 3J1, Canada; [4]MiNA Therapeutics, Translation & Innovation Hub, London W12 0BZ, UK; [5]Department of Chemistry, McGill University, Montreal, Québec H3A 0B8, Canada; [6]Division of Hematology, Department of Medicine, McGill University, Montreal, Québec H4A 3J1, Canada

Acute myeloid leukemia (AML) is a highly aggressive blood cancer marked by impaired differentiation and uncontrolled proliferation of myeloid cells. This phenotype is often driven by dysregulated expression of the transcription factor C/EBPα (encoded by *CEBPA*), especially in high-risk subtypes with *FLT3* mutations. We hypothesized that RNA activation (RNAa) of *CEBPA* could reduce the growth of FLT3-mutated AML, and synergize with currently approved FLT3 inhibitors, thereby offering an alternative treatment strategy for a deadly disease. Our study shows that MTL-CEBPA, a chemically modified small activating RNA encapsulated in NOV340 liposomes, selectively targets myeloid cells, boosts *CEBPA* expression, and promotes a non-proliferative, mature state in FLT3-mutated AML cells. Importantly, MTL-CEBPA enhances the efficacy of commonly prescribed FLT3 inhibitor, gilteritinib, both *in vitro* and *in vivo*. All together, these findings support RNAa of *CEBPA* as a potential adjuvant therapy for FLT3-mutated AML.

INTRODUCTION

Acute myeloid leukemia (AML) is the most common and lethal adult leukemia.[1] The disease is characterized by blocked differentiation and uncontrolled proliferation of immature myeloblasts.[2] The balance between proliferation and differentiation in hematopoiesis is tightly regulated by transcription factors (TFs), which modulate downstream gene expression by interacting with cofactors and binding to promoter/enhancer regions.[3–6] One essential TF regulator of myeloid differentiation is CCAAT/enhancer-binding protein alpha (C/EBPα),[7] which plays a pivotal role in the transition from common myeloid progenitors to granulocyte-monocyte progenitors.[8,9] Under physiological conditions, C/EBPα activity limits myeloid progenitor proliferation[10,11] by inducing cell-cycle exit[12–14] through modulation of cell-cycle-related factors (transcription of p21,[10] repression of E2F,[13] and suppression of c-Myc[15]) and promotes terminal myeloid differentiation[16,17] by upregulating myeloid-specific TFs[18] (e.g., PU.1[19,20] and CEBPε[21,22]) and genes encoding colony-stimulating factor receptors (e.g., *CSF3R*[23–25] and *CSF2RA*[26]). Conversely, loss of C/EBPα activity is associated with block in terminal myeloid differentiation and increased progenitor proliferation[27–29] and self-renewal.[30]

C/EBPα dysregulation plays a significant role in AML: approximately half of cases have a reduction in *CEBPA* mRNA compared to normal myeloid progenitors,[31,32] and loss-of-function mutations have been reported in 5%–10% of patients.[33] Furthermore, C/EBPα is inhibited post-translationally in AML cases that contain mutations in the FMS-like tyrosine kinase 3 (FLT3) receptor (such as internal tandem duplications, ITDs; present in 30% of AML patients[34]). Aberrant FLT3 signaling leads to inhibition of C/EBPα activity by serine 21 phosphorylation via ERK1/2-signaling[35,36] and ubiquitin-mediated degradation.[37] Due to C/EBPα′s critical role in the differentiation-proliferation axis and its chronic inhibition in FLT3-mutated AML, there is a strong rationale to target C/EBPα and upregulate its expression to reduce the uncontrolled growth of AML cells.

TFs have often been referred to as "undruggable" due to their nuclear localization[38] and lack of a ligand binding site.[39] Even though advancements have been made in this field,[40,41] they remain challenging therapeutic targets. Nucleic acid therapeutics (NATs) are well poised to overcome this hurdle.[42,43] By targeting the expression of TFs at the transcriptional or translational levels, NATs can selectively modulate their activity.[44] However, lack of selective delivery approaches greatly limits the therapeutic use of NATs.[45] Further, upregulating the activity of a TF remains challenging.[46] Beyond gene editing systems,[47,48] options to directly increase the

Received 24 December 2024; accepted 11 June 2025;
https://doi.org/10.1016/j.omtn.2025.102611.

Correspondence: Nathan W. Luedtke, Department of Pharmacology & Therapeutics, McGill University, Montreal, Québec H3G 1Y6, Canada.
E-mail: nathan.luedtke@mcgill.ca
Correspondence: François E. Mercier, Division of Clinical and Translational Research, Department of Medicine, McGill University, Montreal, Québec H4A 3J1, Canada.
E-mail: francois.mercier@mcgill.ca
Correspondence: Maureen McKeague, Department of Pharmacology & Therapeutics, McGill University, Montreal, Québec H3G 1Y6, Canada.
E-mail: maureen.mckeague@mcgill.ca

© 2025 The Author(s).
Published by Elsevier Inc. on behalf of The American Society of Gene and Cell Therapy.
This is an open access article under the CC BY license (http://creativecommons.org/licenses/by/4.0/).

expression of a transcription factor are severely limited. A promising approach to upregulate gene expression involves small activating RNA (saRNA), which are short RNA duplexes designed to target the genomic region of a selected gene and thereby activate transcription.[49,50] Through interactions with Argonaute proteins (specifically AGO2) and other co-factors, saRNAs have been reported to induce transcription initiation by interacting and recruiting RNA polymerase II to the promoter site of the target gene and inducing histone modifications favoring transcriptional activation.[51]

Developed by MiNA Therapeutics, MTL-CEBPA is composed of a NOV340 liposome encapsulating a chemically modified 21-nucleotide saRNA duplex capable of specifically upregulating *CEBPA* gene expression (CEBPA-51).[52] Due to its success in decreasing tumor growth and metastasis in hepatocellular carcinoma rodent models,[53] MTL-CEBPA has advanced to clinical trials as a leading example of saRNA-based therapy.[54] Preclinical studies have also demonstrated anti-inflammatory properties of MTL-CEBPA.[55] To date, phase I clinical trials have shown excellent pharmacokinetic and safety properties.[54,56] In this study, we investigated the potential repurposing of MTL-CEBPA for AML. saRNAs have been explored in the context of chronic myeloid leukemia[57,58] but their application to AML remains largely unexplored; in particular, it remained unknown whether NOV340 liposomes can deliver RNA into AML cells. Additionally, it is not known whether the CEBPA-saRNA can transcriptionally activate *CEBPA* in leukemic blasts and if the consequent upregulation would be therapeutically beneficial for AML.

Here, we demonstrate the targeting and upregulating *CEBPA* expression in AML using MTL-CEBPA. We report that NOV340 liposomes can deliver RNA to human AML cells *in vitro* and *in vivo* in patient- and cell-line-derived xenograft models. We also demonstrate that saRNA-induced upregulation of *CEBPA* sensitizes a well-characterized, FLT3-ITD+ AML line, MOLM-14 AML cells, to FLT3 inhibition. Together, our results indicate that RNA activation of *CEBPA* is possible *in vivo* and may be a viable adjunct therapy for FLT3-ITD+ AML. This work demonstrates the importance and exciting potential of utilizing NATs for the treatment of hematological malignancies.

RESULTS

NOV340 liposomes deliver RNA to myeloid leukemia cell lines

Efficient and targeted delivery of NATs is paramount for their therapeutic efficacy. As delivery is the main obstacle for development of NATs,[59] MTL-CEBPA must be delivered to AML cells for there to be any therapeutic application. Therefore, we first characterized uptake into AML cell lines *in vitro* using a variant in which the saRNA is tagged with Cyanine-3 dye, termed Cy3-MTL-CEBPA. Several controls were included to validate the cellular uptake experiments (Figure 1A). Briefly, PBS-treated cells served as an untreated control to account for cellular autofluorescence and establish baseline Cy3 median fluorescence intensity (MFI); cells treated with unencapsulated (or "naked") Cy3-tagged RNA were used to confirm that cleaved cyanine dye did not cross the cell membrane autonomously, as previously reported by Lacroix et al.,[60] and finally, cells treated with "destroyed" Cy3-MTL-CEBPA control demonstrated that intact liposomes are essential for efficient delivery, as stability is known to be sensitive to temperature and pH fluctuations.[61,62] Following overnight incubation, a statistically significant increase in Cy3 fluorescence ($p < 0.001$) was observed in the Cy3-MTL-CEBPA group compared to all controls in all AML cell lines tested (KG1a, THP-1, MOLM-14, PL-21, OCI-AML3, K562, HL-60, OCI-AML2, and MOLM-13) (Figures 1B and S1A). The increase in Cy3 fluorescence was observed as both MFI and an increased percentage of cells labeled as Cy3+ (via gating). As such, a range of 60%–85% uptake was observed in the tested cells, with THP1 having lowest uptake and KG1a having the highest (Figure 1B). We then expanded the panel of cell lines to include PL-21 (10.9%–11.9%), OCI-AML3 (22.6%–25.2%), K562 (31.7%–32.6%), HL-60 (10%–14.3%), OCI-AML2 (6.3%–7.7%), and MOLM-13 (5%–6.6.2%) (Figures S1B and S1C). In all cases, statistically significant increases were seen compared to controls. Furthermore, uptake of Cy3-MTL-CEBPA in myeloid leukemia cells shows both dose- and time-dependent relationships (Figures S2A and S2B). In contrast, no significant increase in Cy3 fluorescence was noted in the T-acute lymphoblastic leukemia cell line, Jurkat (Figures 1B and S1C). These results suggest that NOV340 liposomes may be preferentially taken up into AML cell lines, offering a strategy for selective delivery of RNA to AML.

To validate that the delivery of Cy3-labeled saRNA was mediated by assembled NOV340 liposomes, we exposed cells to an equivalent dose of an unencapsulated (or "naked") Cy3-RNA as well as "destroyed" Cy3-MTL-CEBPA, in which the structural integrity of the liposome was compromised by disassociating the lipid moieties via temporary exposure to high heat and acidic conditions. Following overnight incubation with unencapsulated Cy3-RNA, there was no significant increase in Cy3 fluorescence in the myeloid leukemia cell lines compared to cells treated with PBS (Figures 1B and S1C). When incubating cells with "destroyed" Cy3-MTL-CEBPA, majority of cell lines (THP-1, MOLM-14, PL-21, K562, OCI-AML2, and MOLM-13) had no significant increase in Cy3 fluorescence compared to PBS-treated cells; whereas HL-60, OCI-AML3, and KG1a had a very small, but significant, increase in Cy3 fluorescence compared to PBS (Figures 1B and S1C). Together, the data suggest that the NOV340 liposomes are responsible for the efficient uptake of RNA into myeloid cell lines.

Cy3-MTL-CEBPA is preferentially taken up into myeloid cells via macropinocytosis

Cy3-MTL-CEBPA has been previously shown to be taken up by non-malignant hematopoietic stem and progenitor cells.[63] However, in these studies, the mechanisms dictating NOV340 liposomal uptake and selectivity were not characterized. Therefore, we aimed to explore the uptake mechanism of NOV340 liposomes in AML cells.

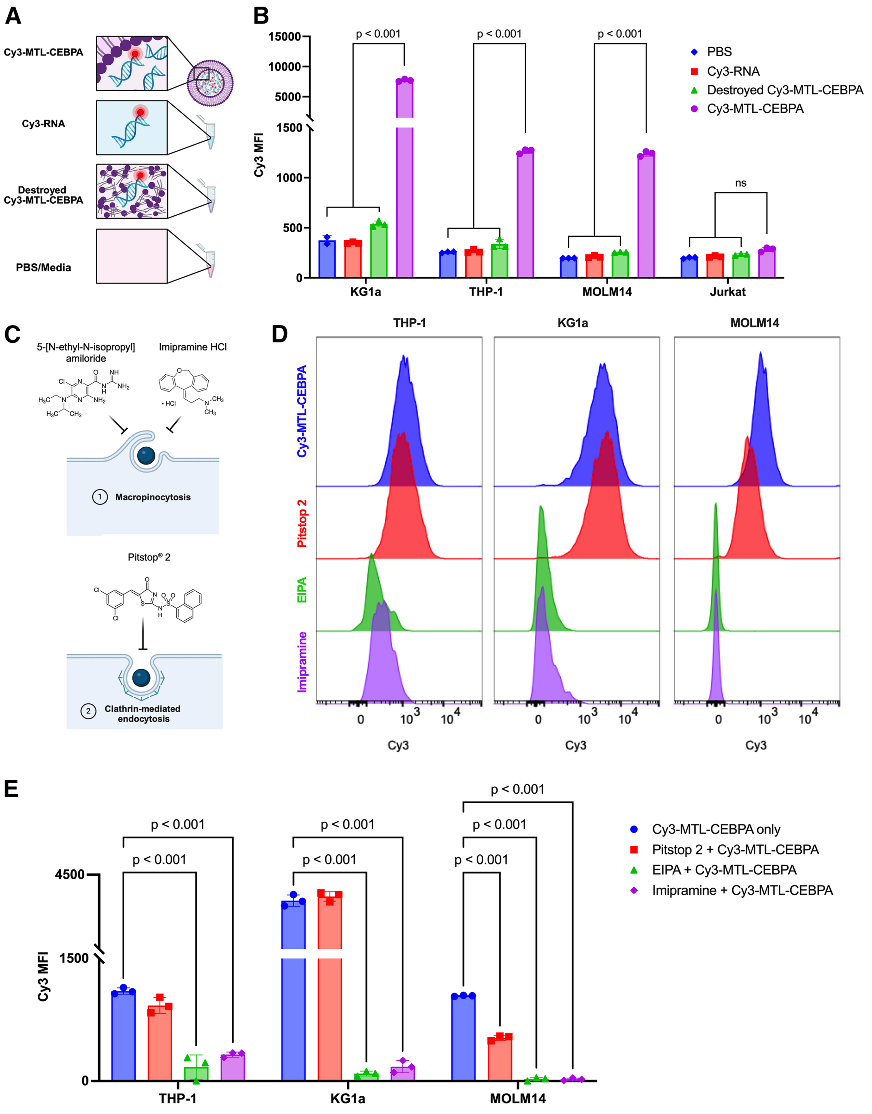

Figure 1. NOV340 liposomes deliver RNA into AML cells *in vitro* via macropinocytosis

(A) Graphic of Cy3-MTL-CEBPA and controls. (B) Delivery of fluorescently tagged CEBPA-51 saRNA in myeloid leukemia and lymphoid leukemia cell lines. Two-way ANOVA with Holm-Sidak multiple comparison test; n = 3. Data represented as mean (SD). (C) Schematic of endocytosis inhibitors used for *in vitro* uptake inhibition assay. (D) Representative histograms of Cy3 fluorescence in AML cell lines after treatment with clathrin-mediated endocytosis inhibitor, Pitstop 2, or macropinocytosis inhibitors, EIPA or imipramine. (E) Changes in uptake of Cy3-MTL-CEBPA following treatment with endocytosis inhibitors in THP-1, MOLM-14, and KG1a cells. Two-way ANOVA with Holm-Sidak multiple comparison test; n = 3. Data represented as mean (SD).

Clathrin-mediated endocytosis (CME) is the most common cellular uptake pathways for liposomes, particularly for liposomes of 50–200 nm in diameter[64,65] such as the NOV340 liposomes (~120 nm in diameter[66,67]). However, given our results demonstrating Cy3-MTL-CEBPA uptake into all myeloid cell lines tested, but not a lymphoid cell line (Jurkat), we hypothesized that the potential myeloid-specific uptake of NOV340 liposomes is mediated by macropinocytosis.[68] In contrast to clathrin-mediated endocytosis, macropinocytosis is a non-specific endocytic mechanism constitutively utilized by myeloid-derived cells (dendritic cells and macrophages) for environment monitoring and antigen presentation.[69] As such, we focused our characterization on comparing the role of CME and macropinocytosis in NOV340 liposomal uptake (Figure 1C).

After treating cells with Pitstop 2 (a validated CME inhibitor that acts by binding to clathrin terminal domains[70]) and Cy3-MTL-CEBPA, THP-1 and KG1a cells showed no significant decrease in Cy3 fluorescence compared to cells without CME inhibitor. In the MOLM-14 cell line, however, inhibition of CME resulted in a small but significant 2-fold reduction in Cy3 fluorescence ($p < 0.001$) (Figures 1D and 1E).

In contrast, following treatment with the macropinocytosis inhibitor EIPA (5-[N-ethyl-N-isopropyl] amiloride),[71] there was a marked reduction in Cy3 fluorescence in all three cell lines. THP-1 showed a 5-fold reduction in Cy3-MTL-CEBPA uptake ($p < 0.001$), whereas MOLM-14 and KG1a cells had 25-fold ($p < 0.001$) and 37-fold ($p < 0.001$) reductions in uptake, respectively. Equivalent inhibition in uptake was observed when the experiment was repeated with an alternative macropinocytosis inhibitor, imipramine[72] (Figures 1D and 1E). Taken together, our results suggest that macropinocytosis is a key mechanism for Cy3-MTL-CEBPA uptake in AML cells and may explain its selective uptake into myeloid cells compared to T cells.

Cy3-MTL-CEPBA accumulates in human AML cells in PDX mouse models

We next aimed to determine whether NOV340 liposomes could deliver RNA to leukemic cells *in vivo*. Previous reports demonstrate that intravenous administration of MTL-CEBPA into wild-type animals results in RNA delivery to non-malignant myeloid cells (monocytes, macrophages, and dendritic cells) in the blood, spleen, and bone marrow.[73] However, it is not yet known if the myeloid cell delivery extends to AML. As such, *in vivo* delivery of Cy3-MTL-CEBPA was tested using patient-derived xenograft (PDX) mouse models.

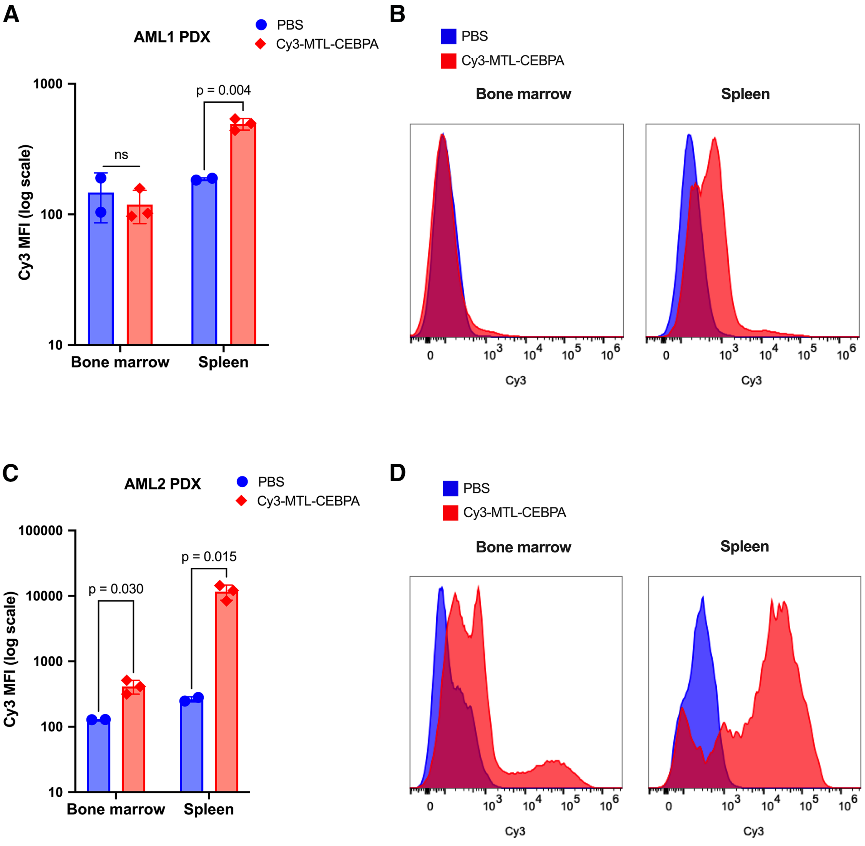

Figure 2. Cy3-MTL-CEBPA is taken up into human leukemic cells *in vivo*

(A) Delivery of Cy3-MTL-CEBPA to hCD45+ leukemic cells in AML1 PDX model. Multiple unpaired student t tests; n = 2–3. Data represented as mean (SD). (B) Representative histogram of Cy3 fluorescence in human CD45+ cells isolated from bone marrow and spleen of AML1 PDX model. (C) Delivery of Cy3-MTL-CEBPA to hCD45+ leukemic cells in AML2 PDX model. Multiple unpaired student t tests; n = 2–3. Data represented as mean (SD). (D) Representative histogram of Cy3 fluorescence in human CD45+ cells isolated from bone marrow and spleen of AML2 PDX model.

In two PDX models (AML1: 50-year-old female with NMP1 mutated, FLT3-ITD+, and CEBPA mutated AML; AML2: 73-year-old male with NMP1 mutated and FLT3-ITD+ AML), 3 mg/kg of Cy3-MTL-CEBPA was injected intravenously, and 4 hours later, the delivery to leukemic cells within the bone marrow and spleen was quantified using Cy3 fluorescence (MFI) (Figure S3). In the first PDX model, AML1, mice injected with Cy3-MTL-CEBPA had no significant increase in Cy3 fluorescence in the human leukemia cells localized to the bone marrow (Figures 2A and 2B). However, Cy3-MTL-CEBPA treatment did result in a significant 2-fold increase in Cy3 fluorescence in human AML cells in the spleen ($p = 0.004$), indicating effective delivery of Cy3-RNA to a proportion of spleen-localized leukemic cells (Figures 2A and 2B).

In the second PDX model tested, AML2, systemic administration of Cy3-MTL-CEBPA resulted in effective RNA delivery into human leukemic cells localized to both the bone marrow and the spleen (Figure 2C). In the bone marrow, a significant portion of human CD45+ cells showed Cy3 fluorescence (3.2-fold increase; $p = 0.030$) (Figure 2D). In the spleen, the majority of leukemic cells exhibited significant Cy3-MTL-CEBPA uptake (44.2-fold increase; $p = 0.015$) (Figure 2D). Interestingly, two distinct populations of human CD45+ cells were identified in the bone marrow, with one group showing a higher propensity for Cy3-MTL-CEBPA uptake. Together, these results suggest that NOV340 liposomes deliver RNA into human leukemic cells in an animal model, but the magnitude of uptake is patient-specific and may be blast-specific within a single patient.

MTL-CEBPA upregulates CEBPA mRNA and C/EBPα protein in AML cells *in vitro*

Having established effective uptake of MTL-CEBPA *in vitro* and *in vivo*, we next aimed to characterize functional RNA activation induced by MTL-CEBPA. Multiple AML cell lines were incubated with MTL-CEBPA or a control, MTL-FLUC, which consists of a firefly luciferase-targeting siRNA encapsulated in the same NOV340 liposomes. Changes in gene expression were analyzed via RT-qPCR 72 h after treatment. Comparing MTL-CEBPA to the control revealed there was an increase in *CEBPA* mRNA across all cell lines tested: 2-fold in THP-1 cells ($p = 0.020$), 1.6-fold in KG1a cells ($p = 0.033$), 2.1-fold in MOLM-14 ($p = 0.009$), 2.5-fold in PL-21 ($p = 0.062$), and 1.9-fold in MOLM-13 ($p = 0.074$) (Figures 3A, S6A, and S7A). The data presented here are reinforced by Zhou et al., who also reported a 1.8-fold increase in *CEBPA* expression in THP-1 cells following transfection with CEBPA-51 saRNA.[55] By normalizing *CEBPA* expression to MTL-FLUC-treated cells, we can conclude that the upregulation is due to the activity of the saRNA pharmacophore, CEBPA-51, and not the NOV340 liposome.

Furthermore, MTL-CEBPA treatment of MOLM-14 cells resulted in a sustained increase in *CEBPA* until day 5, followed by a modest downregulation in expression (Figure S4A). This downregulation is suggested to be due to C/EBPα′s role in autoregulation.[74] At the protein level, an approximate 1.4-fold upregulation in total C/EBPα was observed after MTL-CEBPA treatment in THP-1 and MOLM-14 compared to MTL-FLUC control (Figures 3B, S4B, and S4C).

Importantly, significant *CEBPA* upregulation (3-fold; $p < 0.001$) was observed upon treating a patient sample (named AML3: a 62-year-old male with IDH2, DNMT3A, NMP1, and FLT3-ITD mutated

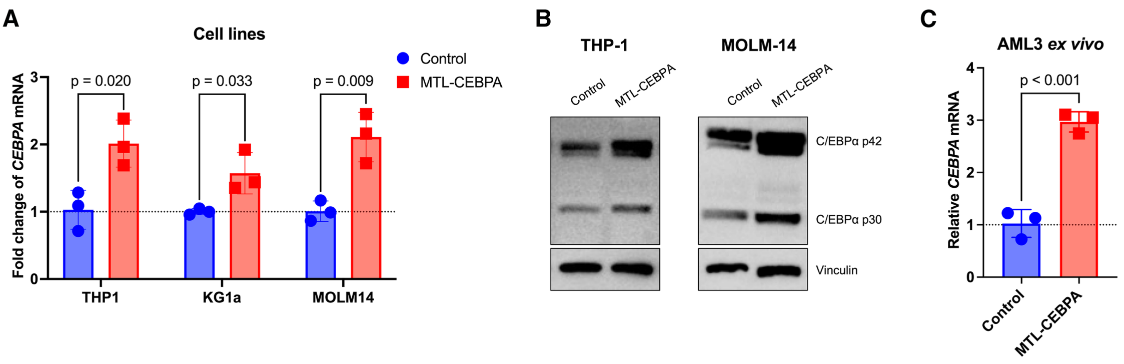

Figure 3. MTL-CEBPA upregulates *CEBPA* in AML *in vitro* models

(A) Changes in *CEBPA* mRNA expression in THP-1, KG1a, or MOLM-14 cell lines 72-h post-MTL-CEBPA or control (MTL-FLUC) treatment. Two-tailed unpaired student t tests; $n = 3$. Data represented as mean (SD). (B) Representative western blots of THP-1 and MOLM-14 cell lines 96 h post-treatment. (C) Upregulation of *CEBPA* mRNA in *ex vivo* AML3 patient sample 48 h post MTL-CEBPA or control (MTL-FLUC) treatment. Two-tailed unpaired student t test; $n = 3$. Data represented as mean (SD).

AML) *ex vivo* with MTL-CEBPA (Figure 3C), supporting that RNA activation via MTL-CEBPA extends beyond immortalized cell lines. Together, these findings confirm the function of CEBPA-51 for activating the TF at both the RNA and protein levels.

Systemic administration of MTL-CEBPA upregulates *CEBPA* expression in an MOLM-14-xenograft mouse model

Given our promising results demonstrating uptake of RNA via liposomes in a mouse PDX model, we sought to explore whether systemic administration of MTL-CEBPA could upregulate *CEBPA* expression in human leukemia cells engrafted in the bone marrow and spleen of mice. Following treatment of the MOLM-14 cell line xenograft model with intravenous injections of MTL-CEBPA or MTL-FLUC control (Figure 4A), the human leukemic cells were harvested from the bone marrow and spleen and isolated via fluorescence-activated cell sorting. The cells isolated from the MTL-CEBPA-treated mice had a 1.8-fold higher expression of *CEBPA* mRNA ($p = 0.008$) (Figure 4B) and 1.4-fold increase in C/EBPα than mice treated with control (Figure 4C).

In the same *in vivo* model, we aimed to assess whether the observed increase in C/EBPα was sufficient to alter downstream effects, particularly the expression of another key regulator of myelomonocytic differentiation: PU.1, encoded by *SPI1* gene, which is a well-known target of C/EBPα[75,76] through direct binding of promoter[19] and/or enhancer regions.[20] Mice treated with MTL-CEBPA had significantly elevated *SPI1* mRNA expression (1.7-fold) compared to mice treated with control ($p = 0.009$) (Figure 4B). Taken together, our results demonstrate that systemic administration of MTL-CEBPA *in vivo* can upregulate *CEBPA* expression and the resultant expression of a downstream target of C/EBPα, *SPI1*, in leukemic cells.

MTL-CEBPA sensitizes MOLM-14 cells to FLT3 inhibitor gilteritinib

C/EBPα is targeted by the malignant FLT3-ITD signaling cascade,[36,77,78] and restoration of C/EBPα activity plays a central role in recovery of myeloid differentiation after FLT3 inhibition.[79] Therefore, we hypothesized that upregulating *CEBPA* using MTL-CEBPA can enhance leukemic cell response to small molecule FLT3 inhibitors. Specifically, we screened eight different FLT3 inhibitors (lestaurtinib, gilteritinib, midostaurin, crenolanib, sorafenib, quizartinib, ponatinib, and sunitinib) in an MOLM-14 cell line engineered to express luciferase and Venus fluorescent proteins (MOLM-14-Ven-Luc) to compare the impact of MTL-CEBPA and FLT3 inhibition on cell growth. These small molecule inhibitors differ based on specificity/selectivity for FLT3 receptor and mechanism of action (type I vs. type II)[76] (Table S1). Type I FLT3 inhibitors (lestaurtinib, gilteritinib, midostaurin, and crenolanib) only bind and inhibit the receptor when it is in an activated conformation and therefore equally target both wild-type and mutated FLT3. Type II FLT3 inhibitors (sorafenib, quizartinib, ponatinib, sunitinib) bind and stabilize the inactive conformation of FLT3-ITD, thereby preventing the activation of the receptor. Given the differing mechanisms, we quantified the concentration that resulted in 50% growth inhibition (IC_{50}) for every inhibitor in the presence of MTL-CEBPA or the control MTL-FLUC.

From the dose-response curves, we found that MTL-CEBPA lowered the IC_{50} for five out of the eight FLT3 inhibitors: gilteritinib, midostaurin, sorafenib, quizartinib, and ponatinib by 17.7-, 6.4-, 13.9-, 5.8-, and 4-fold, respectively (Figure 5A). These results indicate that pretreating cells with MTL-CEBPA improved the potency of these FLT3 inhibitors in MOLM-14-Ven-Luc cells. Based on this preliminary screen, we selected gilteritinib for further investigation due to the enhanced combinatorial effect with MTL-CEBPA and since it is the only approved FLT3 inhibitor for relapsed/refractory AML.[80] After repeating the dose-response assay, MTL-CEBPA in combination with gilteritinib resulted in a 12-fold lower IC_{50} versus cells treated with MTL-FLUC control and gilteritinib ($p < 0.001$) (Figures 5B and S5A), demonstrating that MTL-CEBPA sensitizes MOLM-14-Ven-Luc cells to gilteritinib *in vitro*.

MTL-CEBPA alters growth and maturation of AML cells *in vitro*

To investigate why MTL-CEBPA sensitizes MOLM-14-Ven-Luc cells to gilteritinib, we characterized changes in gene expression,

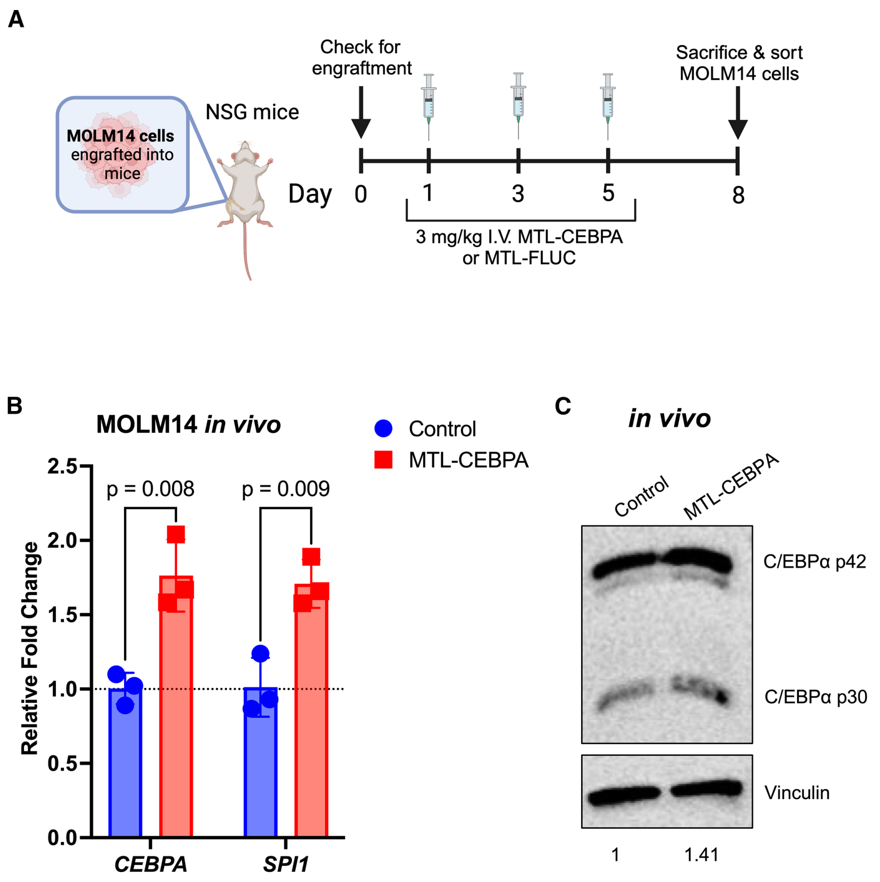

Figure 4. MTL-CEBPA upregulates *CEBPA* following systemic administration in an *in vivo* model of AML

(A) Schematic for *in vivo* upregulation experiment. (B) Change in *CEBPA* and *SPI1* mRNA expression in leukemic cells isolated from MOLM-14-xenograft mouse model. Two-tailed unpaired student t test; $n = 3$ (each data point represents the pooled samples of two mice). Data represented as mean (SD). (C) Western blot of C/EBPα expression in isolated leukemic cells from MOLM14-xenograft mouse model ($n = 1$; each sample is a pool of six mice).

cell growth, and cell surface marker expression following MTL-CEBPA treatment. Multiple reports have demonstrated associations between the differentiation status, proliferative capacity, and gene expression profile of AML blasts and their responsiveness to tyrosine kinase inhibitors. For example, Casado et al. determined that patient-derived AML cells with higher expression of markers of myelomonocytic differentiation were more sensitive to tyrosine kinase inhibition.[81] Moreover, Ma et al. reported that all-*trans* retinoic acid synergizes with sorafenib by inducing differentiation of leukemic cells, thereby rendering them more responsive to FLT3 inhibition.[82] Therefore, we hypothesized that MTL-CEBPA sensitizes AML cells to the FLT3 inhibitor, gilteritinib, by limiting leukemic cell proliferation and promoting myeloid differentiation.

First, we examined changes in cell growth after MTL-CEBPA treatment. MOLM-14 cells treated with MTL-CEBPA demonstrated a significant reduction in cell growth compared to control-treated cells ($p < 0.001$) (Figure 5C). MOLM-14 cells treated with MTL-CEBPA and gilteritinib had a slower growth trend than cells treated with control (MTL-FLUC) and gilteritinib, although not statistically significant ($p = 0.15$) (Figure 5C). Nonetheless, MOLM-14 cells treated with MTL-CEBPA and gilteritinib had significantly lower viability on day 6 after treatment compared to cells treated with control and gilteritinib ($p = 0.002$), indicating enhanced efficacy of the combination treatment (Figure 5D). Similar decreases in cell growth and viability were noted with two other FLT3-ITD-mutated AML cell lines, MOLM-13 (Figures S6B and S6C) and PL-21 (Figures S7B and S7C).

Next, we assessed for changes in c-Myc expression in MOLM-14 cells. c-Myc is a transcription factor and master regulator of cell proliferation and growth and inhibitor of differentiation in many cell types, including the myeloid lineage.[15,83] Hence, the expression of c-Myc strongly correlates with proliferation, acting as a marker for the proliferative capacity of cells. We observed that after treating MOLM-14 cells with MTL-CEBPA, there was a significant decrease over time in c-Myc expression (Figures 5E and 5F), with MOLM-14 cells having >65% reduction in c-Myc expression on day 7 of MTL-CEBPA treatment compared to day 1 ($p < 0.001$). Coupled with the changes in cell growth, these results indicate that MTL-CEBPA treatment induces changes in the proliferation of MOLM-14 cells.

Lastly, we examined the expression of cell surface markers CD11b and CD14 in AML cell lines after treatment with MTL-CEBPA. Both CD11b and CD14 are related to myeloid differentiation, with their expression increasing as cells mature.[84,85] Following treatment with MTL-CEBPA, MOLM-14, MOLM-13, and PL-21 cells had a significant increase in CD11b MFI by 2.9- ($p = 0.041$), 3.2- ($p < 0.001$), and 3-fold ($p = 0.002$), respectively, compared to control (Figures 5G, S6D, and S7D). Similarly, both cell lines demonstrated an increase in CD14 expression following MTL-CEBPA treatment, with MOLM-14, MOLM-13, and PL-21 cells having a 3.3- ($p = 0.049$), 3.4- ($p < 0.001$), and 2.6-fold ($p = 0.002$) increase in CD14 MFI expression, respectively (Figures 5H, S6E, and S7E). In line with the changes in cell surface marker expression, we also note an upregulation of *SPI-1* expression in MOLM-14 ($p = 0.004$), but a downregulation of *FLT3* mRNA ($p = 0.020$), following MTL-CEBPA treatment (Figure S5B), suggesting that C/EBPα may be altering the transcription of genes involved in leukemic cell maturation.

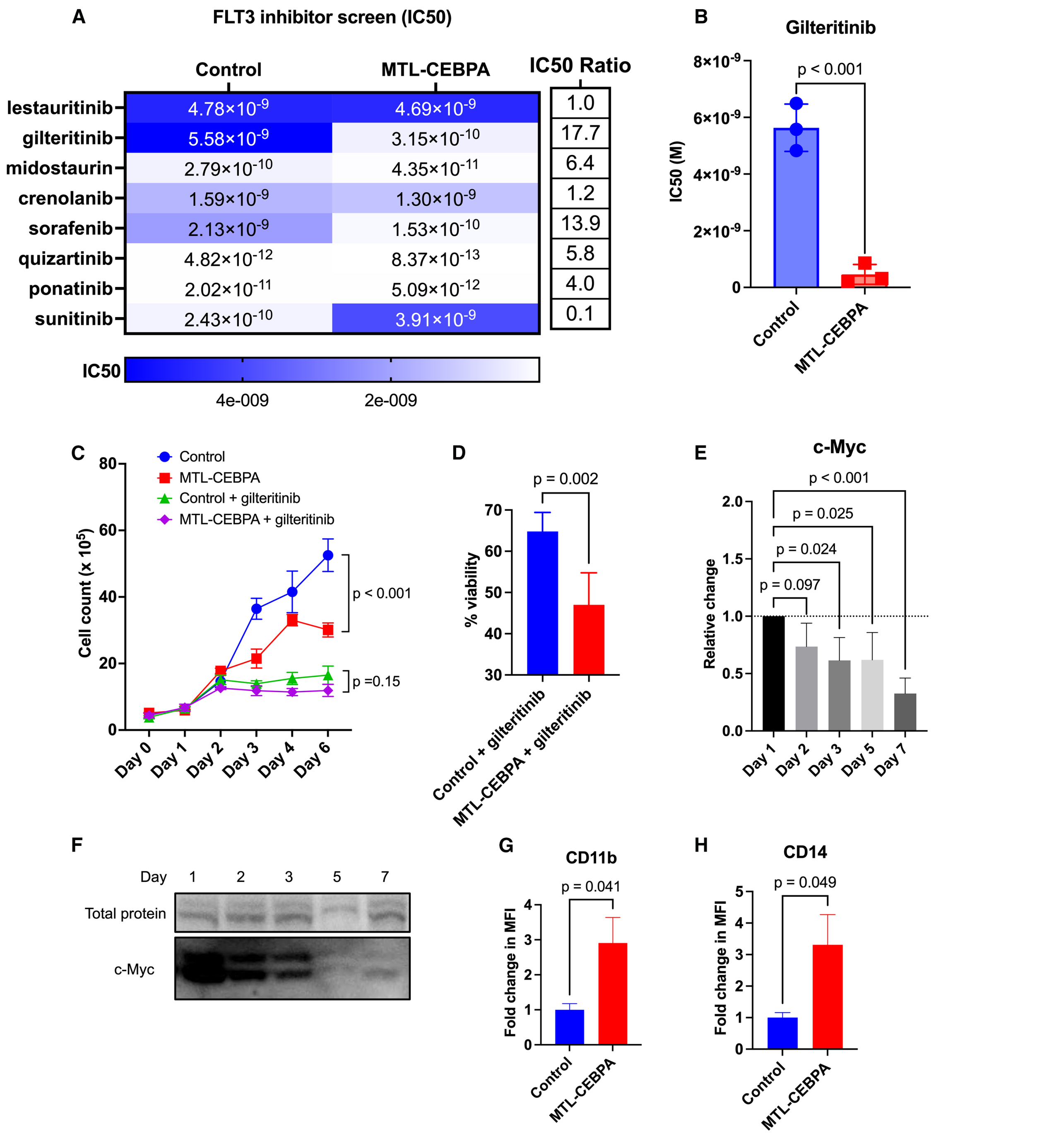

Figure 5. MTL-CEBPA sensitizes MOLM-14 cells to the anti-proliferative effects of FLT3 inhibitors

(A) Heatmap of FLT3 inhibitors showing IC_{50} values when combined with MTL-CEBPA or control (MTL-FLUC) in MOLM-14-Ven-Luc cells. The fold change in the IC50 (control vs. MLT-CEBPA) for each FLT3 inhibitor is listed next to the heatmap. (B) Difference in gilteritinib IC_{50} in MOLM-14-Ven-Luc cells treated with MTL-CEBPA or control (MTL-FLUC). Two-way unpaired student t test; $n = 3$. Data represented as mean (SD). (C) Growth curves for MOLM-14 cells following treatment with 10 μg/mL of MTL-CEBPA or control (MTL-FLUC) with and without 5 nM gilteritinib. Two-way ANOVA with Holm-Sidak multiple comparison test; $n = 6$. Data represented as mean (SEM). (D) Percent viability of cells on day 6. Two-way unpaired student t test; $n = 6$. Data represented as mean (SD). (E) Decrease in c-Myc expression overtime after treatment with 10 μg/mL MTL-CEBPA. One-way ANOVA with Holm-Sidak multiple comparison test; n = 2–3. Data represented as mean (SD). (F) Representative western blot of c-Myc expression over time in MOLM-14 cells. (G) Fold change in CD11b MFI and (H) CD14 MFI in MOLM-14 cells 96 h following treatment with 10 μg/mL MTL-CEBPA or control (untreated). Two-way unpaired student t test; n = 2–3. Data represented as mean (SD).

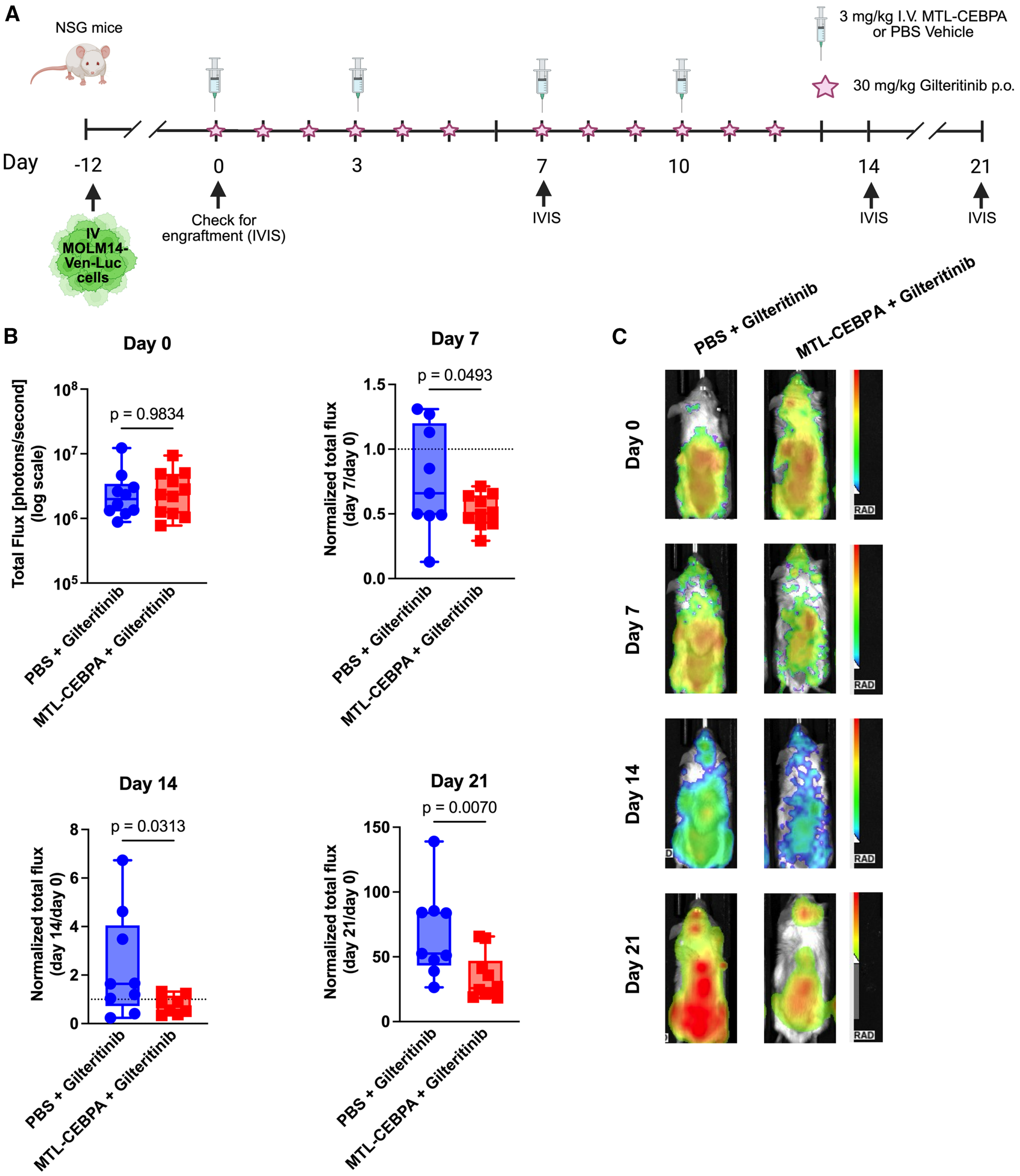

Figure 6. MTL-CEBPA enhances anti-leukemic effects of gilteritinib *in vivo*

(A) Schematic depicting treatment schedule for therapeutic evaluation of MTL-CEBPA in MOLM-14-Ven-Luc cell-line-derived xenotransplantation model. (B) Change in leukemic burden (as measured by luciferase bioluminescence) in MOLM-14-xenograft mice following treatment with MTL-CEBPA + gilteritinib or PBS + gilteritinib. One-tailed unpaired student t test; n = 10–11. (C) Representative images obtained from *in vivo* bioluminescence imaging.

MTL-CEBPA enhances the anti-leukemic effect of gilteritinib *in vivo*

After validating the increased sensitivity of AML cells to gilteritinib upon MTL-CEBPA treatment *in vitro*, we sought to test whether the combination of MTL-CEBPA and gilteritinib could reduce leukemic burden *in vivo* using a xenograft mouse model of AML. In the MOLM-14-Ven-Luc cell-line-derived xenotransplantation model, leukemic burden was measured using luciferase bioluminescence through an *in vivo* imaging system (IVIS),[86] at baseline and weekly thereafter (Figure 6A). Mice were treated for 2 weeks with either MTL-CEBPA + gilteritinib or PBS vehicle + gilteritinib. Seven days into treatment, the MTL-CEBPA + gilteritinib treatment group

displayed a >50% reduction in the amount of detectable leukemic cells, whereas the gilteritinib group showed a lesser (∼25%) reduction in leukemic burden compared to the first day of treatment (p = 0.049). After completing the 14-day treatment period, mice treated with MTL-CEBPA + gilteritinib maintained the leukemic burden below pre-treatment levels (∼25% less leukemia compared to day 0), whereas mice treated with gilteritinib alone had a 230% increase in leukemic burden compared to start of treatment (p = 0.031). Furthermore, we assessed the leukemic burden 1 week after stopping treatment (day 21). Mice treated with the combination treatment had significantly lower levels of leukemic cells (2-fold less) than the mice treated only with gilteritinib (p = 0.007) (Figures 6B and S8A–S8E). Nonetheless, a substantial number of residual AML cells persist in the bone marrow of the mice following treatment. No significant differences were observed between the groups after examining the cell surface marker expression of the residual cells (Figure S9). Together, these results show that MTL-CEBPA enhances the anti-proliferative activity of gilteritinib and delays regrowth of AML cells even after treatment.

DISCUSSION

Despite advances in AML therapy, most targeted treatments fail to produce durable remissions, with relapse common within 1–2 years,[87] highlighting the critical need for new drug combinations with greater efficacy and tolerability. Interestingly, recent pre-clinical studies have shown that the anti-leukemic effect of inhibitors of mutated FLT3 can be enhanced through combination with other targeted agents (e.g., inhibitors of Pim kinases, BET, BCL-2, or oxidative phosphorylation),[88–92] and response to FLT3 inhibition has been linked with myeloid differentiation of AML cells[79,93] and restoration of C/EBPα activity.[77,94] Building upon this concept, we sought to evaluate MTL-CEBPA, a liposomal-formulated saRNA promoting the expression of *CEBPA* and myeloid differentiation, for its ability to enhance the therapeutic effect of FLT3 inhibition. Overall, our results demonstrate that the delivery of CEBPA-51 saRNA into AML cell lines and patient-derived cells *in vivo* is feasible, leading to a significant upregulation of C/EBPα and enhanced therapeutic effect of gilteritinib in an AML mouse model.

RNA activation offers a promising strategy to modulate gene expression, though its clinical application remains complex. Previously shown by Voutila et al., the CEBPA-51 saRNA acts to upregulate *CEBPA* expression by associating with the genomic locus of *CEBPA* via an AGO2- and CTR9-dependent mechanism,[52] which is in line with the mechanism described by Li et al.[51] Here, our results support RNA activation as an efficacious way to harness the transcriptional and translational machinery in cells and increase the expression of hard-to-drug transcription factors. We demonstrate that the delivery of CEBPA-51 saRNA into AML cell lines results in a significant upregulation of *CEBPA* expression. Using a xenograft mouse model, we show that MTL-CEBPA upregulates *CEBPA* expression in AML cells when administered systemically and that this *CEBPA* upregulation results in the downstream upregulation of a C/EBPα-modulated gene, *SPI1*. We also show that *CEPBA* upregulation using MTL-CEBPA can enhance the anti-leukemic effects of gilteritinib and induce a greater reduction in leukemic burden than gilteritinib alone. As C/EBPα acts as a tumor suppressor[95] and restoring its expression results in a better outcome for multiple solid cancers, we hypothesize that this enhanced effect is mediated partly through MTL-CEPBA's ability to reduce leukemic cell proliferation, potentially by promoting myeloid differentiation. These findings demonstrate that direct upregulation of *CEBPA* using RNA activation can sensitize human AML cells to FLT3 inhibition.

Although the precise mechanism by which MTL-CEBPA enhances FLT3 inhibitor efficacy remains to be fully defined, our data show that MTL-CEBPA modulates genes linked to proliferation and differentiation, thereby limiting leukemic growth. Volpe et al. demonstrated that C/EBPα binds *FLT3* intronic enhancers and that both its knockdown and moderate overexpression reduce *FLT3* mRNA, indicating that dysregulation in either direction suppresses *FLT3* expression.[96] Ansari et al. further showed that a ∼30% siRNA-mediated reduction in *FLT3* mRNA enhanced FLT3 inhibitor sensitivity in AML cells.[97] These findings support a model in which CEBPA-mediated *FLT3* reduction sensitizes AML cells to FLT3 inhibitors. Consistently, our preliminary data show that MTL-CEBPA increases *CEBPA* mRNA and modestly but significantly reduces *FLT3* mRNA in MOLM-14 cells. Ongoing work aims to clarify this potential mechanism. Notably, only five out of the eight FLT3 inhibitors demonstrated potential synergism with MTL-CEBPA, specifically gilteritinib, sorafenib, midostaurin, quizartinib, and ponatinib. To explore this, we grouped the compounds based on their clinical relevance. Out of the eight, four (gilteritinib,[80] midostaurin,[98] sorafenib,[99] and quizartinib[100]) are currently used for the treatment of AML. All four demonstrated enhanced effects in combination with MTL-CEBPA (Table S1), highlighting a potential therapeutic window for combination strategies in FLT3-ITD+ AML.

Our findings underscore the therapeutic potential of saRNA in AML while also highlighting key translational hurdles that must be overcome. A critical roadblock toward the application of RNA therapeutics in the clinic is the delivery into specific target cells. We found that the NOV340 liposomes deliver CEBPA-51 saRNA to human AML lines *in vitro* primarily via macropinocytosis, which potentially explains the selective uptake into myeloid cells compared to a T cell line. However, we observed heterogeneity in the uptake *in vitro*, with MOLM-13 cells showing 5% uptake, whereas KG1a cells showed 85%. We also observed this heterogeneity in uptake *in vivo*-, where systemic administration of Cy3-MTL-CEBPA into mice harboring human leukemic blasts revealed altered uptake between two patients and even potentially between leukemic cells within a patient. Therefore, clinical application of NOV340 particles in AML would still require screening of patient samples for efficient uptake. However, there are ongoing efforts to improve liposomal targeting to leukemias,[101–103] whereby the RNA pharmacophore could be repackaged for improved delivery. Future work will involve evaluating therapeutic efficacy in PDX models stratified by uptake efficiency,

which would provide critical insight into the variability of response and guide patient selection criteria for RNA-based therapies.

Another major challenge in AML therapy is disease recurrence driven by residual leukemic stem cells that evade treatment. We recognize that MTL-CEBPA may not effectively eliminate these cells, which represents a significant limitation. Our findings show that following discontinuation of treatment with MTL-CEBPA and gilteritinib, a population of residual leukemic cells remains capable of repopulating the bone marrow. To address this, we propose that MTL-CEBPA (or optimized next-generation analogs) will be most effective as part of a combination treatment regimen targeting FLT3-ITD AML.

Isoform-specific effects of C/EBPα represent another important consideration for MTL-CEBPA's clinical development. Specifically, two distinct C/EBPα isoforms exist: the 42 kDa one (p42), which is capable of both DNA-binding and *trans*-activation activity crucial for regulating genes involved in differentiation, and the 30 kDa C/EBPα (p30), a dominant negative competitor of the 42 kDa isoform that lacks a *trans*-activation domain.[7] Thus, a potential limitation of this approach is that MTL-CEBPA upregulates both isoforms, potentially limiting efficacy for promoting differentiation. A follow-up study in a larger set of patient samples and cell lines is required to assess whether baseline or maximal expression levels of C/EBPα isoforms are reliable markers of biological response. We expect that MTL-CEBPA would not be suitable for *CEBPA*-mutated AML, which occurs *de novo* or occasionally as a resistance mechanism in response to FLT3 inhibition.

Beyond targeting leukemic cells, MTL-CEBPA may also exert immunomodulatory effects with relevance for AML immunotherapy. Interestingly, Plummer et al. report that the combination of MTL-CEBPA and an immune-checkpoint inhibitor (ICI; pembrolizumab) convert immunologically "cold" tumors into "hot" (inflamed) tumor microenvironments by inducing the differentiation of progenitor monocytes into immunoreactive HLA-DR+ myeloid cells that recruit cytotoxic T cell to the tumor site.[56,104] AML is often referred to as a "cold" tumor due to its immunosuppressive microenvironment,[105,106] where immunomodulating drugs such as ICIs have shown limited clinical efficacy.[107] Thus, whether MTL-CEBPA might also alter the immunogenicity of AML should be further tested in immunocompetent models. Supporting this hypothesis, viral transduction of solid tumors with *CEBPA* alone[108] or with other TFs[109] was recently shown to promote MHC-II-driven tumor antigen presentation. As our study has already demonstrated the efficient targeting and delivery of MTL-CEBPA to the bone marrow microenvironment, investigating the immunomodulatory effects of MTL-CEBPA in AML could be a future research avenue to sensitize the AML immune microenvironment to ICIs.

As with every NAT, safety and immunogenicity are critical concerns. The phase I trials of MTL-CEBPA revealed that once a week administration is well tolerated in humans and that it appears to have little-to-no reported toxicity at that administration interval and dose.[54,56] As for immunogenicity, none of the adverse events reported in the phase I trials were consistent with immune activation. Supporting this, *in vitro* studies using human peripheral blood mononuclear cells showed that transfection with the chemically modified saRNA "CEBPA-51" did not elicit significant secretion of the inflammatory cytokines tumor necrosis factor alpha (TNF-α) or interferon alpha (IFN-α) (unpublished data). These results indicate that the saRNA does not activate Toll-like receptor 7/8/9 pathways and therefore lacks immune-stimulatory activity.

In conclusion, our findings provide a proof-of-concept for using saRNA to upregulate transcription factors in AML. We demonstrate that MTL-CEBPA enhances the therapeutic efficacy of FLT3 inhibition *in vivo*, providing a potential strategy for targeting FLT3-ITD+ AML. Further optimization of delivery systems will be essential, but this work sets the stage for advancing RNA activation approaches in hematologic malignancies.

MATERIALS AND METHODS

Chemicals and reagents

MTL-CEBPA (*CEBPA*-targeting saRNA, CEBPA-51, encapsulated in NOV340 liposomes[53,66]; 2 mg/mL in saline; long-term storage at −20°C; once thawed, stored at 4°C for maximum of 5 weeks) and the control liposome-formulated firefly luciferase (FLUC)-targeting siRNA (MTL-FLUC; 2 mg/mL in saline; long-term storage at −20°C) were provided by MiNA Therapeutics, London, UK.[52,54] The prefix "MTL" in "MTL-CEBPA" refers to MiNA Therapeutics Limited, whereas "CEBPA" denotes the target gene of the saRNA, C/EBPα. Cy3-MTL-CEBPA is composed of a Cy3-tagged *CEBPA*-targeting saRNA sequence encapsulated in NOV340 formulation. The lipid components of these liposomes are comprised of l-palmitoyl-2-oleoyl-sn-glycero-3-phosphocholine (POPC); l,2-dioleoyl sn-glycero-3-phosphoethanolamine (DOPE); cholesteryl-hemisuccinate (CHEMS); and 4-(2-aminoethyl)-morpholino-cholesterol hemisuccinate (MOCHOL) at a molar ratio of 6:24:23:47. These liposomes were prepared by crossflow ethanol injection method (∼120 nm in diameter[66,67]). Details regarding composition of MTL-CEBPA can be found in patent entitle "C/EBP ALPHA SARNA COMPOSITIONS AND METHODS OF USE" (WO 2016/170349 Al).

RNA sequences are as follows:

C/EBPα saRNA (CEBPA-51): sense, 5′-GCmG GmUC mAUmU GmUC mACmU GmGU CmUmU-3′ (m: 2′-OMe modified), and antisense, 5′-GAC CAG UGA CAA UGA CCG CmUmU-3′ (m: 2′-OMe modified).

Firefly luciferase (FLUC) siRNA: sense, 5′-mCmUmU AmCG mCmUG AGmU AmCmU mUmCG AdTpsdT-3′ (m: 2′-OMe modified; ps: phosphorothioate), and antisense, 5′-UCG AAG mUAC UmU A GCG mUAA GdTpsdT-3′ (m: 2′-OMe modified; ps: phosphorothioate).

All cell culture products were purchased from Gibco (Gibco-BRL, a division of Life Technologies). Sources for FLT3 inhibitors are as follows: gilteritinib (SelleckChem); sorafenib and midostaurin (Sigma); quizartinib, sunitinib Malate, and lestaurtinib (VWR); crenolanib (Abcam); and ponatinib (Fisher Scientific). Sources for small molecule endocytosis inhibitors include 5-(N-Ethyl-N-isopropyl) amiloride (Sigma-Aldrich); Imipramine HCl (Sigma-Aldrich); and Pitstop2 (Abcam). Upon receipt, all compounds were resuspended in DMSO, aliquoted, and stored at −20°C.

Cell lines

AML cell lines OCI-AML3, OCI-AML2, MOLM-13, MOLM-14, HS-5, and PL-21 cell lines were obtained from the Leibniz Institute DSMZ-German Collection of Cell Cultures GmbH. THP-1, HL-60, K562, and KG1a cell lines were obtained from the American Type Culture Collection (ATCC). THP-1, HS-5, KG1a, PL-21, K562, HL-60, OCI-AML2, MOLM-13, and MOLM-14 cells were maintained in RPMI-1640 supplemented with 10% FBS (fetal bovine serum, Gibco) and 1% Gibco ABAM (Antibiotic-Antimycotic: 100 units/mL of penicillin, 100 μg/mL of streptomycin, and 0.025 μg/mL of Gibco Amphotericin B). OCI-AML3 was maintained in RPMI-1640 supplemented with 20% FBS and 1% ABAM. Cells were cultured in a humidified 5% CO_2 incubator at 37°C and periodically tested for mycoplasma. The MOLM-14-Venus-Luciferase cell line was generated by lentiviral transduction, as previously described.[86]

In vitro drug treatments

Cell viability was determined with Trypan Blue staining (Gibco), and cells were seeded at a concentration of 5×10^5 cells/mL 16–24 h prior to start of treatment. For treatment with MTL-CEBPA or MTL-FLUC, cells were washed once in PBS and then resuspended in serum-free RPMI-1640 with 1% ABAM. MTL-CEBPA or MTL-FLUC was added to culture media for a final concentration of 2 μg/mL, unless otherwise specified. Cells were incubated in serum-free conditions for 6 h, and then the media was supplemented with FBS to a final concentration of 10%. Cells were incubated for various lengths of time depending on the experimental endpoints: 72 or 96 h for RNA extraction or cell surface marker analysis; 96 h for protein extraction; 16 h prior to FLT3 inhibitor treatment; maximum of 144 h for cell growth analysis.

For FLT3 inhibitor treatment, all drugs were diluted in media immediately prior to an experiment to a final concentration of 1.5 mM. Serial dilutions of each FLT3 inhibitor were prepared by diluting the stock concentration using a 1:3 dilution ratio in complete media.

Cell growth curves

MTL-CEBPA- or MTL-FLUC (control)-treated cells were seeded into 96-well plates (one plate per time point per cell line) and treated with 5 nM gilteritinib or media. On days 0 (seeding day), 1, 2, 3, 4, and 6, cells were counted using the Spark multimode reader (Tecan) and cell chip adaptor. On day 6, cell viability was determined with Trypan Blue staining (Gibco).

Cy3-MTL-CEBPA *in vitro* uptake

Cell viability was determined with Trypan Blue staining (Gibco), and cells were seeded at a concentration of 5×10^5 cells/mL in 6-well plates 16–24 h prior to start of treatment. Cells were washed once in PBS and resuspended in serum-free RPMI-1640 with 1% ABAM. Cy3-MTL-CEBPA or a control (Cy3 Transfection Control DsiRNA [Integrated DNA technologies], heat/acid-destroyed Cy3-MTL-CEBPA, or PBS) was added to culture media for a final concentration of 2 μg/mL, unless otherwise specified, and incubated overnight (∼16 h). Cells were then collected, washed twice with PBS, and stained for live and dead cells with the LIVE/DEAD fixable green dead cell stain kit (Thermo Fisher Scientific) according to the manufacturer's instructions. Flow cytometry analysis was then performed on samples using the Sony ID7000 Full spectrum flow cytometer with a minimum of 10,000/sample events collected. Results were analyzed using the FlowJo software (version 10.10.0, FlowJo LLC), in which live cells were gated, and the median fluorescence intensity (MFI) of Cy3 was obtained. Supplemental Methods are available in the SI.

Endocytic inhibitor assay

To assess the uptake mechanism of Cy3-MTL-CEBPA, cells were treated with either macropinocytosis inhibitors, 0.25 mM 5-(N-Ethyl-N-isopropyl) amiloride or 0.1 mM Imipramine HCl, or clathrin-mediated endocytosis inhibitor, 3 μM Pitstop 2, for 1 h prior to the addition of Cy3-MTL-CEBPA to cell culture media. Samples were then incubated for 4 h with 2 μg/mL Cy3-MTL-CEBPA in serum-free conditions at 37°C. Cells were harvested as described above, washed twice with 2% FBS/PBS, and stained with the LIVE/DEAD fixable green dead cell stain kit (Thermo Fisher Scientific) according to the manufacturer's instructions. Samples were analyzed using the Sony ID7000 Full spectrum flow cytometer, and the median fluorescence intensity was obtained using FlowJo software (version 10.10.0, FlowJo LLC).

MOLM-14-Venus-Luciferase *in vitro* cell proliferation assay

5×10^4 cells/well in 96-well plates were treated with either 2 μg/mL of MTL-CEBPA or MTL-FLUC in serum-free media for 6 h and then supplemented with serum (final concentration 10%) and left to recover overnight. The next morning, the cells were treated with 10 different concentrations of FLT3 inhibitors (12.7 pM–0.75 μM) for 72 hours at 37°C and 5% CO_2. Venus fluorescence was used as an indirect measure of cell proliferation. Fluorescence was quantified using a Tecan Spark microplate reader, and data were normalized to wells containing only media. Non-linear regression analysis was performed to obtain the concentration at which 50% of cells stop proliferating (IC_{50}).

RNA quantification

Total RNA was extracted with Monarch Total RNA Miniprep Kit (New England BioLabs) from at least 500,000 cells and quantified using a BioSpectrometer Fluorescence (Eppendorf) according to manufacturer's instruction. GoScript Reverse Transcription Mix, Oligo

(dT) (Promega) was used for reverse transcription of 5–20 ng/mL of RNA to cDNA in a 20 μL reaction.

2x TaqMan Fast Advanced Master Mix (Thermo Fisher Scientific) and 60x TaqMan Gene Expression Assays (Thermo Fisher Scientific) were used for real-time quantitative PCR analysis to quantify expression levels of *CEBPA* (Hs00269972_s1), *SPI1* (Hs02786711_m1), *FLT3* (Hs00174690_m1), *GAPDH* (Hs02786624_g1), and *HPRT1* (Hs02800695_m1). qPCR was conducted using CFX96 Real-Time System (Bio-Rad) and C1000 Touch Thermal Cycler (Bio-Rad). Relative expression was determined using the ΔΔCt method normalized to *GAPDH* and *HPRT1* expression.

Western blot

Minimum of 3×10^6 cells were lysed using ice-cold RIPA buffer (50 mM Tris, 150 mM NaCl, 0.1% SDS, 0.5% deoxycholate, 1% Triton X-100) supplemented with protease inhibitor and centrifuged for 15 min at 16,000 × g at 4° C. Pierce BCA Protein Assay kit (Thermo Fisher Scientific) was used to quantify protein according to the manufacturer's protocol. Equal volume of 2x Laemmli sample buffer (4% SDS, 20% glycerol, 0.004% bromophenol blue, 0.125 M Tris-Cl, pH 6.8, 10% 2-mercaptoethanol) was mixed with protein sample and heated at 70°C for 10 min. Twenty microgram of total protein was loaded onto 10% separating and 6% stacking SDS-polyacrylamide gel. Protein expression was confirmed by 4° C overnight incubation of PVDF membrane containing protein of interest with C/EBPα (Rabbit, polyclonal, 1:1,000, Cell Signaling Technology), c-Myc (Rabbit, polyclonal, 1:1,000, Cell Signaling Technology), or Vinculin (Rabbit, monoclonal, 1:1,000; Cell Signaling Technology, clone E1E9V) followed by 1-h incubation with anti-rabbit HRP-conjugated secondary antibody (1:2,000; New England BioLabs) at room temperature. Immunoblots were visualized by Pierce ECL Western Blotting Substrate (Thermo Fisher Scientific) according to the manufacturer's protocol, and the chemiluminescent signals were captured using CCD camera-based imager, Amersham Imager 600 system (GE Healthcare Life Sciences). Relative integrated density values were calculated by Fiji ImageJ 1.53c (National Institutes of Health) and GraphPad Prism 9.4.1.

Culturing patient samples

All experiments were performed in accordance with the regulations of the Institutional Review Board of the Jewish General Hospital [11–047]. All donors provided written, informed consent.

Primary cell samples were thawed and transferred in a stepwise manner over 3 min to a 50 mL falcon tube containing 5 mL of pre-warmed complete media (20% FBS/1% ABAM in RPMI-1640). Afterward, 20 mL of complete media was added, and the cells were centrifuged for 5 min at 300 × g and resuspended in 10 mL media. Cell viability was determined with Trypan Blue staining (Gibco) and analyzed by Tecan Spark microplate reader. Cells were then used for either PDX mouse model generation or *ex vivo* treatments. For the *ex vivo* treatment with MTL-CEBPA/MTL-FLUC, primary cells were co-cultured with the feeder cell line HS-5. As such, the HS-5 cells were seeded in 6-well plates at a density of 1×10^5 cells/mL per well 24 h prior to seeding of the primary blood cells at a density of 5×10^5 cells/mL per well. The primary samples (AML3 sample was from 62-year-old male with IDH2, DNMT3A, NMP1, and FLT3-ITD mutated AML) were incubated for 48 h prior to mRNA analysis.

In vivo patient-derived xenograft mouse model

The PDX models used in this study were generated using adult male NSGS mice (NOD-SCID; IL2Rγ null; Tg(IL3, CSF2, KITL), The Jackson Laboratory), which were treated with 20 mg/kg of busulfan intraperitonially (i.p.) ≤24 h before transplant (2×10^6 cells, intravenous [i.v.] injection). The AML1 PDX sample was from a 50-year-old female with NMP1-mutated, FLT3-ITD+, and CEBPA-mutated AML, whereas the AML2 PDX sample was from a 73-year-old male with NMP1-mutated and FLT3-ITD+ AML. Engraftment was confirmed using bone marrow aspirate, as previously described.[110]

For *in vivo* uptake experiment, mice were intravenously injected with 3 mg/kg of Cy3-MTL-CEBPA or PBS and sacrificed 4 h following injection. The femur, tibia, and spleen were removed and crushed using mortar and pestle. The red blood cells were lysed using ACK lysis buffer (Gibco) and resuspended in 10% FBS/PBS. The samples were then stained for human CD45 marker (Brilliant Violet 421, mouse, monoclonal, 1:100, BioLegend, clone HI30) and analyzed using the Sony ID7000 full-spectrum flow cytometer. Using the FlowJo software (version 10.10.0, FlowJo LLC), hCD45+ Cy3+ cells were gated.

In vivo MOLM-14-xenograft mouse model

All *in vivo* experiments were approved by the animal care committee of McGill University. All mice were housed at the animal care facilities and had ad libitum water and food access and were maintained on a 12-h light/day cycle, mean temperature 22.5 ± 1.5°C, and 22%–28% humidity. Mice were weighed daily. Eight- to twelve-week-old male NOD.Cg-Prkdc^{scid}IL2Rg^{tm1Wjil}/SzJ (NSG) mice were i.p. injected with 20 mg/kg of busulfan 1 day prior to transplant and given enrofloxacin in their drinking water for 14 days. Venus- and luciferase-expressing MOLM-14 cells were washed in complete media (10% FBS/1% ABAM in RPMI-1640), re-suspended in complete media at 1×10^6 cells/mL, and 200 μL (2×10^5 cells) i.v. injected. Mice were assessed for engraftment at 12 days post-transplant by bioluminescence imaging using the Ami HT optical imaging system. For *in vivo* upregulation of *CEBPA,* mice were treated with three intravenous injections of 3 mg/kg MTL-CEBPA or MTL-FLUC over a period of 5 days (Figure 4A). To investigate the therapeutic potential of MTL-CEBPA and gilteritinib, mice were treated with two cycles of 6-day treatments (30 mg/kg gilteritinib p.o. daily; 3 mg/kg MTL-CEBPA or PBS i.v. every 3rd day) with 1 day rest in between the cycles (Figure 6A). MTL-FLUC (siRNA-targeting firefly luciferase) could not be used as a control for measuring changes in leukemic burden due to interference with the *in vivo* bioluminescence readout. Powder gilteritinib (SelleckChem) was resuspended in DMSO and then diluted in 0.5% methylcellulose, with a maximum final concentration of 10% DMSO. MTL-CEBPA/MTL-FLUC was provided in saline

solution (0.9% NaCl) and diluted using PBS. Mice were sacrificed if >20% weight loss occurred or moribund.

In vivo bioluminescence imaging

At 12 days post-transplant, NSG mice were given one i.p. injection of 150 mg/kg D-luciferin in PBS, as previously described.[86] Briefly, mice were anesthetized with 2% isoflurane and imaged using the Ami HT optical imaging system. Images were captured using Spectral Viewer software, and total flux values (photons/second) were determined using regions of interest (ROIs) of the same size for each mouse. Mice were imaged on the days indicated in Figures 4A and 6A to assess engraftment of Venus- and luciferase-expressing MOLM-14 cells. The final total flux value for each mouse was calculated by normalizing to the day 0 value (day X/day 0).

Cell sorting

Mononuclear cells were purified from whole-mouse bone marrow using Ficoll-Paque density centrifugation. Venus-positive cells were sorted using FACS Aria Fusion (Becton Dickinson) and collected for RNA extraction.

Flow cytometry

For *in vitro* assays, drug-treated cells were centrifugated at 600 × g for 5 min, and the pellet was washed twice with PBS supplemented with 2% FBS (2% FBS/PBS). Anti-human CD11b antibody (Brilliant Ultra Violet 496, mouse, monoclonal, 1:1000, eBioscience, clone ICRF44) or anti-human CD14 antibody (Brilliant Violet 421, mouse, monoclonal, BioLegend, clone HCD14) was diluted in 2% FBS/PBS to the manufacturer-recommended concentration, and cells were incubated on ice in the dark with 100 μL of the solution for 30 min. Samples from all conditions were washed and stained for live and dead cells with either LIVE/DEAD Fixable Green Dead Cell Stain kit (Thermo Fisher Scientific) or Zombie NIR Fixable Viability kit (Biolegend) according to the manufacturer's instructions.

Statistical analysis

Unless otherwise noted, error bars in all figures represent SD. GraphPad Prism software (Version 10.3.1) was used for statistical analyses, and differences were considered statistically significant when $p < 0.05$. All datasets were tested for outliers using a ROUT's statistical outlier test. Outliers were removed prior to statistical analyses.

DATA AVAILABILITY

All data associated with this study are present in the paper or the supplemental information. Additional data related to this paper may be requested from the authors.

ACKNOWLEDGMENTS

O.K. is supported by a Canadian Cancer Society Research Training Award: PhD level (CCS award #708370) and previously by a CIHR Doctoral Canada Graduate Scholarship, NSERC PROMOTE Graduate Award, and a CRBS Studentship Award from the Centre de recherche en biologie structural (funded by Fonds de Recherche du Québec, Health Sector, Research Centres Grant #288558). Support for the project includes initial seed funding from MiNA Therapeutics (research agreement), the Canada Foundation for Innovation's (John R. Evans Leaders Fund to M.M.), and the Cole Foundation (Transition Award to M.M.), as well as ongoing funding from CIHR (Grant CIHR PJT-186077 to M. M., N.W.L., and F.E.M.). M.M. is supported through the Canada Research Chairs Program, F.E.M. is an FRQS Junior 2 Clinical Research Scholar, and N.W.L. is supported as a James McGill Professor. M.M. and N.W.L. are members of the Center for RNA Sciences. Some experiments were performed with the McGill University Imaging and Molecular Biology Platform (IMBP) equipment or services. We thank the Flow Cytometry Core Facility and the Imaging and Phenotyping Core Facility at the Lady Davis Institute for their help and training.

AUTHOR CONTRIBUTIONS

Conceptualization, M.M., N.W.L., and F.E.M. Experimental implementation, O.K., B.S. A., and C.G.L. Experimental design and analysis, O.K., M.M., N.W.L., F.E.M., B.M.R., and V.R. Writing (original draft), O.K. and M.M. Writing (review & editing), O.K., M.M., F.E.M., N.W.L., B.M.R., and V.R. All authors reviewed and approved the final version of the manuscript.

DECLARATION OF INTERESTS

V.R. and B.M.R. are employees and shareholders of MiNA Therapeutics Limited. N.W. L., M.M., F.E.M., and O.K. are inventors on a patent related to this work filed by MiNA Therapeutics Limited (WO/2024/175887, issued 29 August 2024). The remaining authors have no competing interests.

DECLARATION OF GENERATIVE AI AND AI-ASSISTED TECHNOLOGIES IN THE WRITING PROCESS

During the preparation of this work, the author(s) used ChatGPT4 to refine sentence structure. After using this tool/service, the author(s) reviewed and edited the content as needed and take(s) full responsibility for the content of the publication.

SUPPLEMENTAL INFORMATION

Supplemental information can be found online at https://doi.org/10.1016/j.omtn.2025.102611.

REFERENCES

1. De Kouchkovsky, I., and Abdul-Hay, M. (2016). Acute myeloid leukemia: a comprehensive review and 2016 update. Blood Cancer J. *6*, e441. https://doi.org/10.1038/bcj.2016.50.
2. Wachter, F., and Pikman, Y. (2024). Pathophysiology of Acute Myeloid Leukemia. Acta Haematol. *147*, 229–246. https://doi.org/10.1159/000536152.
3. Wang, Z., Wang, P., Li, Y., Peng, H., Zhu, Y., Mohandas, N., and Liu, J. (2021). Interplay between cofactors and transcription factors in hematopoiesis and hematological malignancies. Signal Transduct. Target. Ther. *6*, 24. https://doi.org/10.1038/s41392-020-00422-1.
4. Benyoucef, A., Haigh, J.J., and Brand, M. (2023). Unveiling the complexity of transcription factor networks in hematopoietic stem cells: implications for cell therapy and hematological malignancies. Front. Oncol. *13*, 1151343. https://doi.org/10.3389/fonc.2023.1151343.
5. Orkin, S.H. (1995). Transcription Factors and Hematopoietic Development. J. Biol. Chem. *270*, 4955–4958. https://doi.org/10.1074/jbc.270.10.4955.
6. Corces, M.R., Buenrostro, J.D., Wu, B., Greenside, P.G., Chan, S.M., Koenig, J.L., Snyder, M.P., Pritchard, J.K., Kundaje, A., Greenleaf, W.J., et al. (2016). Lineage-specific and single-cell chromatin accessibility charts human hematopoiesis and leukemia evolution. Nat. Genet. *48*, 1193–1203. https://doi.org/10.1038/ng.3646.
7. Friedman, A.D. (2015). C/EBPα in normal and malignant myelopoiesis. Int. J. Hematol. *101*, 330–341. https://doi.org/10.1007/s12185-015-1764-6.
8. Tenen, D.G. (2003). Disruption of differentiation in human cancer: AML shows the way. Nat. Rev. Cancer *3*, 89–101. https://doi.org/10.1038/nrc989.
9. Friedman, A.D. (2007). Transcriptional control of granulocyte and monocyte development. Oncogene *26*, 6816–6828. https://doi.org/10.1038/sj.onc.1210764.
10. Timchenko, N.A., Wilde, M., Nakanishi, M., Smith, J.R., and Darlington, G.J. (1996). CCAAT/enhancer-binding protein alpha (C/EBP alpha) inhibits cell

proliferation through the p21 (WAF-1/CIP-1/SDI-1) protein. Genes Dev. *10*, 804–815. https://doi.org/10.1101/gad.10.7.804.

11. Wang, H., Iakova, P., Wilde, M., Welm, A., Goode, T., Roesler, W.J., and Timchenko, N.A. (2001). C/EBPalpha arrests cell proliferation through direct inhibition of Cdk2 and Cdk4. Mol. Cell *8*, 817–828. https://doi.org/10.1016/s1097-2765(01)00366-5.
12. Porse, B.T., Pedersen, T.A., Xu, X., Lindberg, B., Wewer, U.M., Friis-Hansen, L., and Nerlov, C. (2001). E2F repression by C/EBPalpha is required for adipogenesis and granulopoiesis in vivo. Cell *107*, 247–258. https://doi.org/10.1016/s0092-8674(01)00516-5.
13. Slomiany, B.A., D'Arigo, K.L., Kelly, M.M., and Kurtz, D.T. (2000). C/EBPalpha inhibits cell growth via direct repression of E2F-DP-mediated transcription. Mol. Cell Biol. *20*, 5986–5997. https://doi.org/10.1128/mcb.20.16.5986-5997.2000.
14. Timchenko, N.A., Wilde, M., and Darlington, G.J. (1999). C/EBPalpha regulates formation of S-phase-specific E2F-p107 complexes in livers of newborn mice. Mol. Cell Biol. *19*, 2936–2945. https://doi.org/10.1128/mcb.19.4.2936.
15. Pelengaris, S., Khan, M., and Evan, G. (2002). c-MYC: more than just a matter of life and death. Nat. Rev. Cancer *2*, 764–776. https://doi.org/10.1038/nrc904.
16. Friedman, A.D., Keefer, J.R., Kummalue, T., Liu, H., Wang, Q.-f., and Cleaves, R. (2003). Regulation of granulocyte and monocyte differentiation by CCAAT/enhancer binding protein α. Blood Cells Mol. Dis. *31*, 338–341. https://doi.org/10.1016/S1079-9796(03)00135-9.
17. Scott, L.M., Civin, C.I., Rorth, P., and Friedman, A.D. (1992). A novel temporal expression pattern of three C/EBP family members in differentiating myelomonocytic cells. Blood *80*, 1725–1735. https://doi.org/10.1182/blood.V80.7.1725.1725.
18. Friedman, A.D. (2002). Transcriptional regulation of granulocyte and monocyte development. Oncogene *21*, 3377–3390. https://doi.org/10.1038/sj.onc.1205324.
19. Kummalue, T., and Friedman, A.D. (2003). Cross-talk between regulators of myeloid development: C/EBPalpha binds and activates the promoter of the PU.1 gene. J. Leukoc. Biol. *74*, 464–470. https://doi.org/10.1189/jlb.1202622.
20. Yeamans, C., Wang, D., Paz-Priel, I., Torbett, B.E., Tenen, D.G., and Friedman, A. D. (2007). C/EBPalpha binds and activates the PU.1 distal enhancer to induce monocyte lineage commitment. Blood *110*, 3136–3142. https://doi.org/10.1182/blood-2007-03-080291.
21. Shyamsunder, P., Shanmugasundaram, M., Mayakonda, A., Dakle, P., Teoh, W.W., Han, L., Kanojia, D., Lim, M.C., Fullwood, M., An, O., et al. (2019). Identification of a novel enhancer of CEBPE essential for granulocytic differentiation. Blood *133*, 2507–2517. https://doi.org/10.1182/blood.2018886077.
22. Theilgaard-Mönch, K., Pundhir, S., Reckzeh, K., Su, J., Tapia, M., Furtwängler, B., Jendholm, J., Jakobsen, J.S., Hasemann, M.S., Knudsen, K.J., et al. (2022). Transcription factor-driven coordination of cell cycle exit and lineage-specification in vivo during granulocytic differentiation : In memoriam Professor Niels Borregaard. Nat. Commun. *13*, 3595. https://doi.org/10.1038/s41467-022-31332-1.
23. Zhang, P., Iwama, A., Datta, M.W., Darlington, G.J., Link, D.C., and Tenen, D.G. (1998). Upregulation of interleukin 6 and granulocyte colony-stimulating factor receptors by transcription factor CCAAT enhancer binding protein alpha (C/EBP alpha) is critical for granulopoiesis. J. Exp. Med. *188*, 1173–1184. https://doi.org/10.1084/jem.188.6.1173.
24. Iida, S., Watanabe-Fukunaga, R., Nagata, S., and Fukunaga, R. (2008). Essential role of C/EBPα in G-CSF-induced transcriptional activation and chromatin modification of myeloid-specific genes. Genes Cells *13*, 313–327. https://doi.org/10.1111/j.1365-2443.2008.01173.x.
25. Kato, N., Kitaura, J., Doki, N., Komeno, Y., Watanabe-Okochi, N., Togami, K., Nakahara, F., Oki, T., Enomoto, Y., Fukuchi, Y., et al. (2011). Two types of C/EBPα mutations play distinct but collaborative roles in leukemogenesis: lessons from clinical data and BMT models. Blood *117*, 221–233. https://doi.org/10.1182/blood-2010-02-270181.
26. Hohaus, S., Petrovick, M.S., Voso, M.T., Sun, Z., Zhang, D.E., and Tenen, D.G. (1995). PU.1 (Spi-1) and C/EBP alpha regulate expression of the granulocyte-macrophage colony-stimulating factor receptor alpha gene. Mol. Cell Biol. *15*, 5830–5845. https://doi.org/10.1128/mcb.15.10.5830.
27. Porse, B.T., Bryder, D., Theilgaard-Mönch, K., Hasemann, M.S., Anderson, K., Damgaard, I., Jacobsen, S.E.W., and Nerlov, C. (2005). Loss of C/EBP alpha cell cycle control increases myeloid progenitor proliferation and transforms the neutrophil granulocyte lineage. J. Exp. Med. *202*, 85–96. https://doi.org/10.1084/jem.20050067.
28. Seipel, K., Marques, M.T., Bozzini, M.-A., Meinken, C., Mueller, B.U., and Pabst, T. (2016). Inactivation of the p53–KLF4–CEBPA Axis in Acute Myeloid Leukemia. Clin. Cancer Res. *22*, 746–756. https://doi.org/10.1158/1078-0432.Ccr-15-1054.
29. Reckzeh, K., and Cammenga, J. (2010). Molecular mechanisms underlying deregulation of C/EBPα in acute myeloid leukemia. Int. J. Hematol. *91*, 557–568. https://doi.org/10.1007/s12185-010-0573-1.
30. Zhang, P., Iwasaki-Arai, J., Iwasaki, H., Fenyus, M.L., Dayaram, T., Owens, B.M., Shigematsu, H., Levantini, E., Huettner, C.S., Lekstrom-Himes, J.A., et al. (2004). Enhancement of hematopoietic stem cell repopulating capacity and self-renewal in the absence of the transcription factor C/EBP alpha. Immunity *21*, 853–863. https://doi.org/10.1016/j.immuni.2004.11.006.
31. Musialik, E., Bujko, M., Kober, P., Grygorowicz, M.A., Libura, M., Przestrzelska, M., Juszczyński, P., Borg, K., Florek, I., Jakóbczyk, M., et al. (2014). Comparison of promoter DNA methylation and expression levels of genes encoding CCAAT/enhancer binding proteins in AML patients. Leuk. Res. *38*, 850–856. https://doi.org/10.1016/j.leukres.2014.04.013.
32. Pabst, T., Mueller, B.U., Harakawa, N., Schoch, C., Haferlach, T., Behre, G., Hiddemann, W., Zhang, D.-E., and Tenen, D.G. (2001). AML1–ETO downregulates the granulocytic differentiation factor C/EBPα in t(8;21) myeloid leukemia. Nat. Med. *7*, 444–451. https://doi.org/10.1038/86515.
33. Taube, F., Georgi, J.A., Kramer, M., Stasik, S., Middeke, J.M., Röllig, C., Krug, U., Krämer, A., Scholl, S., Hochhaus, A., et al. (2022). CEBPA mutations in 4708 patients with acute myeloid leukemia: differential impact of bZIP and TAD mutations on outcome. Blood *139*, 87–103. https://doi.org/10.1182/blood.2020009680.
34. Daver, N., Schlenk, R.F., Russell, N.H., and Levis, M.J. (2019). Targeting FLT3 mutations in AML: review of current knowledge and evidence. Leukemia *33*, 299–312. https://doi.org/10.1038/s41375-018-0357-9.
35. Ross, S.E., Radomska, H.S., Wu, B., Zhang, P., Winnay, J.N., Bajnok, L., Wright, W. S., Schaufele, F., Tenen, D.G., and MacDougald, O.A. (2004). Phosphorylation of C/EBPalpha inhibits granulopoiesis. Mol. Cell Biol. *24*, 675–686. https://doi.org/10.1128/mcb.24.2.675-686.2004.
36. Radomska, H.S., Bassères, D.S., Zheng, R., Zhang, P., Dayaram, T., Yamamoto, Y., Sternberg, D.W., Lokker, N., Giese, N.A., Bohlander, S.K., et al. (2006). Block of C/EBP alpha function by phosphorylation in acute myeloid leukemia with FLT3 activating mutations. J. Exp. Med. *203*, 371–381. https://doi.org/10.1084/jem.20052242.
37. Gu, X., Zahran, Z.A.M., Sakre, N., Schuerger, C., and Saunthararajah, Y. (2022). FLT3-ITD Degrades CEBPA Via the Ubiquitin-Proteosome Pathway. Blood *140*, 2993–2994. https://doi.org/10.1182/blood-2022-166612.
38. Dang, C.V., Reddy, E.P., Shokat, K.M., and Soucek, L. (2017). Drugging the 'undruggable' cancer targets. Nat. Rev. Cancer *17*, 502–508. https://doi.org/10.1038/nrc.2017.36.
39. Lu, H., Zhou, Q., He, J., Jiang, Z., Peng, C., Tong, R., and Shi, J. (2020). Recent advances in the development of protein-protein interactions modulators: mechanisms and clinical trials. Signal Transduct. Target. Ther. *5*, 213. https://doi.org/10.1038/s41392-020-00315-3.
40. Bushweller, J.H. (2019). Targeting transcription factors in cancer — from undruggable to reality. Nat. Rev. Cancer *19*, 611–624. https://doi.org/10.1038/s41568-019-0196-7.
41. Henley, M.J., and Koehler, A.N. (2021). Advances in targeting 'undruggable' transcription factors with small molecules. Nat. Rev. Drug Discov. *20*, 669–688. https://doi.org/10.1038/s41573-021-00199-0.
42. Kulkarni, J.A., Witzigmann, D., Thomson, S.B., Chen, S., Leavitt, B.R., Cullis, P.R., and van der Meel, R. (2021). The current landscape of nucleic acid therapeutics. Nat. Nanotechnol. *16*, 630–643. https://doi.org/10.1038/s41565-021-00898-0.
43. Hofman, C.R., and Corey, D.R. (2024). Targeting RNA with synthetic oligonucleotides: Clinical success invites new challenges. Cell Chem. Biol. *31*, 125–138. https://doi.org/10.1016/j.chembiol.2023.09.005.

44. Belgrad, J., Fakih, H.H., and Khvorova, A. (2024). Nucleic Acid Therapeutics: Successes, Milestones, and Upcoming Innovation. Nucleic Acid Ther. *34*, 52–72. https://doi.org/10.1089/nat.2023.0068.

45. Juliano, R.L. (2016). The delivery of therapeutic oligonucleotides. Nucleic Acids Res. *44*, 6518–6548. https://doi.org/10.1093/nar/gkw236.

46. Khorkova, O., Stahl, J., Joji, A., Volmar, C.-H., and Wahlestedt, C. (2023). Amplifying gene expression with RNA-targeted therapeutics. Nat. Rev. Drug Discov. *22*, 539–561. https://doi.org/10.1038/s41573-023-00704-7.

47. Becskei, A. (2020). Tuning up Transcription Factors for Therapy. Molecules *25*, 1902. https://doi.org/10.3390/molecules25081902.

48. Heidersbach, A.J., Dorighi, K.M., Gomez, J.A., Jacobi, A.M., and Haley, B. (2023). A versatile, high-efficiency platform for CRISPR-based gene activation. Nat. Commun. *14*, 902. https://doi.org/10.1038/s41467-023-36452-w.

49. Janowski, B.A., Younger, S.T., Hardy, D.B., Ram, R., Huffman, K.E., and Corey, D. R. (2007). Activating gene expression in mammalian cells with promoter-targeted duplex RNAs. Nat. Chem. Biol. *3*, 166–173. https://doi.org/10.1038/nchembio860.

50. Li, L.-C., Okino, S.T., Zhao, H., Pookot, D., Place, R.F., Urakami, S., Enokida, H., and Dahiya, R. (2006). Small dsRNAs induce transcriptional activation in human cells. Proc. Natl. Acad. Sci. USA *103*, 17337–17342. https://doi.org/10.1073/pnas.0607015103.

51. Portnoy, V., Lin, S.H.S., Li, K.H., Burlingame, A., Hu, Z.-H., Li, H., and Li, L.-C. (2016). saRNA-guided Ago2 targets the RITA complex to promoters to stimulate transcription. Cell Res. *26*, 320–335. https://doi.org/10.1038/cr.2016.22.

52. Voutila, J., Reebye, V., Roberts, T.C., Protopapa, P., Andrikakou, P., Blakey, D.C., Habib, R., Huber, H., Saetrom, P., Rossi, J.J., and Habib, N.A. (2017). Development and Mechanism of Small Activating RNA Targeting CEBPA, a Novel Therapeutic in Clinical Trials for Liver Cancer. Mol. Ther. *25*, 2705–2714. https://doi.org/10.1016/j.ymthe.2017.07.018.

53. Reebye, V., Huang, K.-W., Lin, V., Jarvis, S., Cutilas, P., Dorman, S., Ciriello, S., Andrikakou, P., Voutila, J., Saetrom, P., et al. (2018). Gene activation of CEBPA using saRNA: preclinical studies of the first in human saRNA drug candidate for liver cancer. Oncogene *37*, 3216–3228. https://doi.org/10.1038/s41388-018-0126-2.

54. Sarker, D., Plummer, R., Meyer, T., Sodergren, M.H., Basu, B., Chee, C.E., Huang, K.W., Palmer, D.H., Ma, Y.T., Evans, T.R.J., et al. (2020). MTL-CEBPA, a Small Activating RNA Therapeutic Upregulating C/EBP-α, in Patients with Advanced Liver Cancer: A First-in-Human, Multicenter, Open-Label, Phase I Trial. Clin. Cancer Res. *26*, 3936–3946. https://doi.org/10.1158/1078-0432.Ccr-20-0414.

55. Zhou, J., Li, H., Xia, X., Herrera, A., Pollock, N., Reebye, V., Sodergren, M.H., Dorman, S., Littman, B.H., Doogan, D., et al. (2019). Anti-inflammatory Activity of MTL-CEBPA, a Small Activating RNA Drug, in LPS-Stimulated Monocytes and Humanized Mice. Mol. Ther. *27*, 999–1016. https://doi.org/10.1016/j.ymthe.2019.02.018.

56. Plummer, R., Sodergren, M.H., Hodgson, R., Ryan, B.M., Raulf, N., Nicholls, J.P., Reebye, V., Voutila, J., Sinigaglia, L., Meyer, T., et al. (2025). TIMEPOINT, a phase 1 study combining MTL-CEBPA with pembrolizumab, supports the immunomodulatory effect of MTL-CEBPA in solid tumors. Cell Rep. Med. *6*, 102041. https://doi.org/10.1016/j.xcrm.2025.102041.

57. Su, R., Li, C., Wang, X., Li, Z., Wen, Z., Yin, Z., Huang, G., Liu, Y., Yang, J., Hu, H., et al. (2023). *PPFIA1*-targeting miR-181a mimic and saRNA overcome imatinib resistance in BCR-ABL1-independent chronic myeloid leukemia by suppressing leukemia stem cell regeneration. Mol. Ther. Nucleic Acids *32*, 729–742. https://doi.org/10.1016/j.omtn.2023.04.026.

58. Su, R., Wen, Z., Zhan, X., Long, Y., Wang, X., Li, C., Su, Y., and Fei, J. (2024). Small RNA activation of CDH13 expression overcome BCR-ABL1-independent imatinib-resistance and their signaling pathway studies in chronic myeloid leukemia. Cell Death Dis. *15*, 615. https://doi.org/10.1038/s41419-024-07006-9.

59. Roberts, T.C., Langer, R., and Wood, M.J.A. (2020). Advances in oligonucleotide drug delivery. Nat. Rev. Drug Discov. *19*, 673–694. https://doi.org/10.1038/s41573-020-0075-7.

60. Lacroix, A., Vengut-Climent, E., de Rochambeau, D., and Sleiman, H.F. (2019). Uptake and Fate of Fluorescently Labeled DNA Nanostructures in Cellular Environments: A Cautionary Tale. ACS Cent. Sci. *5*, 882–891. https://doi.org/10.1021/acscentsci.9b00174.

61. Kamiya, M., Matsumoto, M., Yamashita, K., Izumi, T., Kawaguchi, M., Mizukami, S., Tsurumaru, M., Mukai, H., and Kawakami, S. (2022). Stability Study of mRNA-Lipid Nanoparticles Exposed to Various Conditions Based on the Evaluation between Physicochemical Properties and Their Relation with Protein Expression Ability. Pharmaceutics *14*, 2357. https://doi.org/10.3390/pharmaceutics14112357.

62. Paramasivam, P., Franke, C., Stöter, M., Höijer, A., Bartesaghi, S., Sabirsh, A., Lindfors, L., Arteta, M.Y., Dahlén, A., Bak, A., et al. (2022). Endosomal escape of delivered mRNA from endosomal recycling tubules visualized at the nanoscale. J. Cell Biol. *221*, e202110137. https://doi.org/10.1083/jcb.202110137.

63. Hodgson, R.I., Natalia, V., Konstantinos, B., Victoria, D., Bruno, Raulf, N., Habib, R., Koch, T., Reebye, V., Haslett, L., and Ryan, B. (2024). Keystone Symposia's Delivery of Nucleic Acid Therapeutics Conference (Banff).

64. Rejman, J., Oberle, V., Zuhorn, I.S., and Hoekstra, D. (2004). Size-dependent internalization of particles via the pathways of clathrin- and caveolae-mediated endocytosis. Biochem. J. *377*, 159–169. https://doi.org/10.1042/bj20031253.

65. Sousa de Almeida, M., Susnik, E., Drasler, B., Taladriz-Blanco, P., Petri-Fink, A., and Rothen-Rutishauser, B. (2021). Understanding nanoparticle endocytosis to improve targeting strategies in nanomedicine. Chem. Soc. Rev. *50*, 5397–5434. https://doi.org/10.1039/d0cs01127d.

66. Bader, A.G. (2012). miR-34 - a microRNA replacement therapy is headed to the clinic. Front. Genet. *3*, 120. https://doi.org/10.3389/fgene.2012.00120.

67. Simonson, B., and Das, S. (2015). MicroRNA Therapeutics: the Next Magic Bullet? Mini Rev. Med. Chem. *15*, 467–474. https://doi.org/10.2174/1389557515666150324123208.

68. Lin, X.P., Mintern, J.D., and Gleeson, P.A. (2020). Macropinocytosis in Different Cell Types: Similarities and Differences. Membranes *10*, 177. https://doi.org/10.3390/membranes10080177.

69. Yoshida, S., Hoppe, A.D., Araki, N., and Swanson, J.A. (2009). Sequential signaling in plasma-membrane domains during macropinosome formation in macrophages. J. Cell Sci. *122*, 3250–3261. https://doi.org/10.1242/jcs.053207.

70. von Kleist, L., Stahlschmidt, W., Bulut, H., Gromova, K., Puchkov, D., Robertson, M.J., MacGregor, K.A., Tomilin, N., Pechstein, A., Chau, N., et al. (2011). Role of the Clathrin Terminal Domain in Regulating Coated Pit Dynamics Revealed by Small Molecule Inhibition. Cell *146*, 471–484. https://doi.org/10.1016/j.cell.2011.06.025.

71. Masereel, B., Pochet, L., and Laeckmann, D. (2003). An overview of inhibitors of Na(+)/H(+) exchanger. Eur. J. Med. Chem. *38*, 547–554. https://doi.org/10.1016/s0223-5234(03)00100-4.

72. Lin, H.-P., Singla, B., Ghoshal, P., Faulkner, J.L., Cherian-Shaw, M., O'Connor, P. M., She, J.-X., Belin de Chantemele, E.J., and Csányi, G. (2018). Identification of novel macropinocytosis inhibitors using a rational screen of Food and Drug Administration-approved drugs. Br. J. Pharmacol. *175*, 3640–3655. https://doi.org/10.1111/bph.14429.

73. Kwok, A. R., Nina; R., Vikash; Habib, R.; Catley, M.; Blakey, D.; Rossi, J.; Habib, N.. in 15th Annual Meeting of the Oligonucleotide Therapeutics Society (Munich, Germany, 2019).

74. Sunami, Y., Yoshino, S., Yamazaki, Y., Iwamoto, T., and Nakamura, T. (2024). Rapid increase of C/EBPα p42 induces growth arrest of acute myeloid leukemia (AML) cells by Cop1 deletion in Trib1-expressing AML. Leukemia *38*, 2585–2597. https://doi.org/10.1038/s41375-024-02430-4.

75. Koschmieder, S., Rosenbauer, F., Steidl, U., Owens, B.M., and Tenen, D.G. (2005). Role of transcription factors C/EBPalpha and PU.1 in normal hematopoiesis and leukemia. Int. J. Hematol. *81*, 368–377. https://doi.org/10.1532/ijh97.05051.

76. Pundhir, S., Bratt Lauridsen, F.K., Schuster, M.B., Jakobsen, J.S., Ge, Y., Schoof, E. M., Rapin, N., Waage, J., Hasemann, M.S., and Porse, B.T. (2018). Enhancer and Transcription Factor Dynamics during Myeloid Differentiation Reveal an Early Differentiation Block in Cebpa null Progenitors. Cell Rep. *23*, 2744–2757. https://doi.org/10.1016/j.celrep.2018.05.012.

77. Choudhary, C., Schwäble, J., Brandts, C., Tickenbrock, L., Sargin, B., Kindler, T., Fischer, T., Berdel, W.E., Müller-Tidow, C., and Serve, H. (2005). AML-associated Flt3 kinase domain mutations show signal transduction differences compared with Flt3 ITD mutations. Blood *106*, 265–273. https://doi.org/10.1182/blood-2004-07-2942.

78. Mizuki, M., Schwable, J., Steur, C., Choudhary, C., Agrawal, S., Sargin, B., Steffen, B., Matsumura, I., Kanakura, Y., Böhmer, F.D., et al. (2003). Suppression of myeloid transcription factors and induction of STAT response genes by AML-specific Flt3 mutations. Blood *101*, 3164–3173. https://doi.org/10.1182/blood-2002-06-1677.

79. Sexauer, A., Perl, A., Yang, X., Borowitz, M., Gocke, C., Rajkhowa, T., Thiede, C., Frattini, M., Nybakken, G.E., Pratz, K., et al. (2012). Terminal myeloid differentiation in vivo is induced by FLT3 inhibition in FLT3/ITD AML. Blood *120*, 4205–4214. https://doi.org/10.1182/blood-2012-01-402545.

80. Perl, A.E., Martinelli, G., Cortes, J.E., Neubauer, A., Berman, E., Paolini, S., Montesinos, P., Baer, M.R., Larson, R.A., Ustun, C., et al. (2019). Gilteritinib or Chemotherapy for Relapsed or Refractory *FLT3*-Mutated AML. N. Engl. J. Med. *381*, 1728–1740. https://doi.org/10.1056/NEJMoa1902688.

81. Casado, P., Wilkes, E.H., Miraki-Moud, F., Hadi, M.M., Rio-Machin, A., Rajeeve, V., Pike, R., Iqbal, S., Marfa, S., Lea, N., et al. (2018). Proteomic and genomic integration identifies kinase and differentiation determinants of kinase inhibitor sensitivity in leukemia cells. Leukemia *32*, 1818–1822. https://doi.org/10.1038/s41375-018-0032-1.

82. Ma, H.S., Greenblatt, S.M., Shirley, C.M., Duffield, A.S., Bruner, J.K., Li, L., Nguyen, B., Jung, E., Aplan, P.D., Ghiaur, G., et al. (2016). All-trans retinoic acid synergizes with FLT3 inhibition to eliminate FLT3/ITD+ leukemia stem cells in vitro and in vivo. Blood *127*, 2867–2878. https://doi.org/10.1182/blood-2015-05-646786.

83. Johansen, L.M., Iwama, A., Lodie, T.A., Sasaki, K., Felsher, D.W., Golub, T.R., and Tenen, D.G. (2001). c-Myc is a critical target for c/EBPalpha in granulopoiesis. Mol. Cell Biol. *21*, 3789–3806. https://doi.org/10.1128/mcb.21.11.3789-3806.2001.

84. Gupta, D., Shah, H.P., Malu, K., Berliner, N., and Gaines, P. (2014). Differentiation and characterization of myeloid cells. Curr. Protoc. Immunol. *104*, 22f.25.21–22F.5.28. https://doi.org/10.1002/0471142735.im22f05s104.

85. Rosmarin, A.G., Weil, S.C., Rosner, G.L., Griffin, J.D., Arnaout, M.A., and Tenen, D.G. (1989). Differential Expression of CDllb/CD18 (Mol) and Myeloperoxidase Genes During Myeloid Differentiation. Blood *73*, 131–136. https://doi.org/10.1182/blood.V73.1.131.131.

86. Fooks, K., Galicia-Vazquez, G., Gife, V., Schcolnik-Cabrera, A., Nouhi, Z., Poon, W.W.L., Luo, V., Rys, R.N., Aloyz, R., Orthwein, A., et al. (2022). EIF4A inhibition targets bioenergetic homeostasis in AML MOLM-14 cells in vitro and in vivo and synergizes with cytarabine and venetoclax. J. Exp. Clin. Cancer Res. *41*, 340. https://doi.org/10.1186/s13046-022-02542-8.

87. Short, N.J., Konopleva, M., Kadia, T.M., Borthakur, G., Ravandi, F., DiNardo, C.D., and Daver, N. (2020). Advances in the Treatment of Acute Myeloid Leukemia: New Drugs and New Challenges. Cancer Discov. *10*, 506–525. https://doi.org/10.1158/2159-8290.Cd-19-1011.

88. Chen, M., Shen, C., Chen, Y., Chen, Z., Zhou, K., Chen, Y., Li, W., Zeng, C., Qing, Y., Wu, D., et al. (2024). Metformin synergizes with gilteritinib in treating FLT3-mutated leukemia via targeting PLK1 signaling. Cell Rep. Med. *5*, 101645. https://doi.org/10.1016/j.xcrm.2024.101645.

89. Lee, J.K., Chatterjee, A., Scarpa, M., Bailey, C.M., Niyongere, S., Singh, P., Mustafa Ali, M.K., Kapoor, S., Wang, Y., Silvestri, G., and Baer, M.R. (2024). Pim Kinase Inhibitors Increase Gilteritinib Cytotoxicity in FLT3-ITD Acute Myeloid Leukemia Through GSK-3β Activation and c-Myc and Mcl-1 Proteasomal Degradation. Cancer Res. Commun. *4*, 431–445. https://doi.org/10.1158/2767-9764.Crc-23-0379.

90. Lee, L., Hizukuri, Y., Severson, P., Powell, B., Zhang, C., Ma, Y., Narahara, M., Sumi, H., Hernandez, D., Rajkhowa, T., et al. (2021). A novel combination regimen of BET and FLT3 inhibition for FLT3-ITD acute myeloid leukemia. Haematologica *106*, 1022–1033. https://doi.org/10.3324/haematol.2020.247346.

91. Ma, J., Zhao, S., Qiao, X., Knight, T., Edwards, H., Polin, L., Kushner, J., Dzinic, S. H., White, K., Wang, G., et al. (2019). Inhibition of Bcl-2 Synergistically Enhances the Antileukemic Activity of Midostaurin and Gilteritinib in Preclinical Models of FLT3-Mutated Acute Myeloid Leukemia. Clin. Cancer Res. *25*, 6815–6826. https://doi.org/10.1158/1078-0432.Ccr-19-0832.

92. Qiao, X., Ma, J., Knight, T., Su, Y., Edwards, H., Polin, L., Li, J., Kushner, J., Dzinic, S.H., White, K., et al. (2021). The combination of CUDC-907 and gilteritinib shows promising in vitro and in vivo antileukemic activity against FLT3-ITD AML. Blood Cancer J. *11*, 111. https://doi.org/10.1038/s41408-021-00502-7.

93. McMahon, C.M., Canaani, J., Rea, B., Sargent, R.L., Qualtieri, J.N., Watt, C.D., Morrissette, J.J.D., Carroll, M., and Perl, A.E. (2019). Gilteritinib induces differentiation in relapsed and refractory FLT3-mutated acute myeloid leukemia. Blood Adv. *3*, 1581–1585. https://doi.org/10.1182/bloodadvances.2018029496.

94. Zheng, R., Friedman, A.D., Levis, M., Li, L., Weir, E.G., and Small, D. (2004). Internal tandem duplication mutation of FLT3 blocks myeloid differentiation through suppression of C/EBPα expression. Blood *103*, 1883–1890. https://doi.org/10.1182/blood-2003-06-1978.

95. Lourenço, A.R., and Coffer, P.J. (2017). A tumor suppressor role for C/EBPα in solid tumors: more than fat and blood. Oncogene *36*, 5221–5230. https://doi.org/10.1038/onc.2017.151.

96. Volpe, G., Walton, D.S., Del Pozzo, W., Garcia, P., Dassé, E., O'Neill, L.P., Griffiths, M., Frampton, J., and Dumon, S. (2013). C/EBPα and MYB regulate FLT3 expression in AML. Leukemia *27*, 1487–1496. https://doi.org/10.1038/leu.2013.23.

97. Ansari, A.S., Kucharski, C., Kc, R., Nisakar, D., Rahim, R., Jiang, X., Brandwein, J., and Uludağ, H. (2024). Lipopolymer/siRNA complexes engineered for optimal molecular and functional response with chemotherapy in FLT3-mutated acute myeloid leukemia. Acta Biomater. *188*, 297–314. https://doi.org/10.1016/j.actbio.2024.08.053.

98. Stone, R.M., Mandrekar, S.J., Sanford, B.L., Laumann, K., Geyer, S., Bloomfield, C. D., Thiede, C., Prior, T.W., Döhner, K., Marcucci, G., et al. (2017). Midostaurin plus Chemotherapy for Acute Myeloid Leukemia with a *FLT3* Mutation. N. Engl. J. Med. *377*, 454–464. https://doi.org/10.1056/NEJMoa1614359.

99. Burchert, A., Bug, G., Fritz, L.V., Finke, J., Stelljes, M., Röllig, C., Wollmer, E., Wäsch, R., Bornhäuser, M., Berg, T., et al. (2020). Sorafenib Maintenance After Allogeneic Hematopoietic Stem Cell Transplantation for Acute Myeloid Leukemia With FLT3–Internal Tandem Duplication Mutation (SORMAIN). J. Clin. Oncol. *38*, 2993–3002. https://doi.org/10.1200/jco.19.03345.

100. Erba, H.P., Montesinos, P., Kim, H.-J., Patkowska, E., Vrhovac, R., Žák, P., Wang, P.-N., Mitov, T., Hanyok, J., Kamel, Y.M., et al. (2023). Quizartinib plus chemotherapy in newly diagnosed patients with FLT3-internal-tandem-duplication-positive acute myeloid leukaemia (QuANTUM-First): a randomised, double-blind, placebo-controlled, phase 3 trial. Lancet *401*, 1571–1583. https://doi.org/10.1016/S0140-6736(23)00464-6.

101. Jiang, X., Bugno, J., Hu, C., Yang, Y., Herold, T., Qi, J., Chen, P., Gurbuxani, S., Arnovitz, S., Strong, J., et al. (2016). Eradication of Acute Myeloid Leukemia with FLT3 Ligand–Targeted miR-150 Nanoparticles. Cancer Res. *76*, 4470–4480. https://doi.org/10.1158/0008-5472.Can-15-2949.

102. Jyotsana, N., Sharma, A., Chaturvedi, A., Budida, R., Scherr, M., Kuchenbauer, F., Lindner, R., Noyan, F., Sühs, K.W., Stangel, M., et al. (2019). Lipid nanoparticle-mediated siRNA delivery for safe targeting of human CML in vivo. Ann. Hematol. *98*, 1905–1918. https://doi.org/10.1007/s00277-019-03713-y.

103. Lin, A.Y., Rink, J.S., Yang, E., Small, S., Serrano, F., Abaza, Y., Altman, J.K., Platanias, L.C., Thaxton, C.S., and Gordon, L.I. (2022). Synthetic Lipid Nanoparticles Actively Target Acute Myeloid Leukemia (AML) Cells and Induce Ferroptosis through Decreased Expression of Glutathione Peroxidase 4. Blood *140*, 5967–5968. https://doi.org/10.1182/blood-2022-168154.

104. Plummer, R., Sodergren, M., Ryan, B., Tchakov, I., Raulf, N., Hodgson, R., Tan, C., Nicholls, J.P., Adderkin, A., Vasileiadou, N., et al. (2023). Abstract LB192: MTL-CEBPA in combination with pembrolizumab converts an immune desert to an inflamed TME in solid tumors resistant to checkpoint blockade. Cancer Res. *83*, LB192. https://doi.org/10.1158/1538-7445.Am2023-lb192.

105. Serroukh, Y., Hébert, J., Busque, L., Mercier, F., Rudd, C.E., Assouline, S., Lachance, S., and Delisle, J.-S. (2023). Blasts in context: the impact of the immune environment on acute myeloid leukemia prognosis and treatment. Blood Rev. *57*, 100991. https://doi.org/10.1016/j.blre.2022.100991.

106. van Galen, P., Hovestadt, V., Wadsworth Ii, M.H., Hughes, T.K., Griffin, G.K., Battaglia, S., Verga, J.A., Stephansky, J., Pastika, T.J., Lombardi Story, J., et al. (2019). Single-Cell RNA-Seq Reveals AML Hierarchies Relevant to Disease Progression and Immunity. Cell *176*, 1265–1281.e24. https://doi.org/10.1016/j.cell.2019.01.031.

107. Tettamanti, S., Pievani, A., Biondi, A., Dotti, G., and Serafini, M. (2022). Catch me if you can: how AML and its niche escape immunotherapy. Leukemia *36*, 13–22. https://doi.org/10.1038/s41375-021-01350-x.

108. Linde, M.H., Fan, A.C., Köhnke, T., Trotman-Grant, A.C., Gurev, S.F., Phan, P., Zhao, F., Haddock, N.L., Nuno, K.A., Gars, E.J., et al. (2023). Reprogramming Cancer into Antigen-Presenting Cells as a Novel Immunotherapy. Cancer Discov. *13*, 1164–1185. https://doi.org/10.1158/2159-8290.Cd-21-0502.

109. Ascic, E., Åkerström, F., Sreekumar Nair, M., Rosa, A., Kurochkin, I., Zimmermannova, O., Catena, X., Rotankova, N., Veser, C., Rudnik, M., et al. (2024). In vivo dendritic cell reprogramming for cancer immunotherapy. Science *386*, eadn9083. https://doi.org/10.1126/science.adn9083.

110. Chung, Y.R., Kim, E., and Abdel-Wahab, O. (2014). Femoral bone marrow aspiration in live mice. J. Vis. Exp. *89*, 51660. https://doi.org/10.3791/51660.

OMTN, Volume *36*

Supplemental information

RNA activation of *CEBPA*

improves leukemia treatment

Olivia Kovecses, Bahram Sharif-Askari, Cristobal Gonzalez-Losada, Vikash Reebye, Bríd M. Ryan, Nathan W. Luedtke, François E. Mercier, and Maureen McKeague

SUPPLEMENTAL METHODS

Cy3-MTL-CEBPA dose-uptake assay

Cells were seeded at a concentration of 5 x 10^5 cells/mL 24 hours prior to start of treatment. Cells were washed once in PBS and resuspend in serum-free RPMI-1640 with 1% ABAM. Cy3-MTL-CEBPA was added to culture media for a final concentration of 0 μg/mL, 0.025 μg/mL, 0.050 μg/mL, 0.01 μg/mL, 0.2 μg/mL, 0.4 μg/mL, 0.8 μg/mL, 1.6 μg/mL, 3.125 μg/mL, 6.25 μg/mL, 12.5 μg/mL, or 25 μg/mL. Cells were left in Cy3-MTL-CEBPA-containing media overnight (~16 hours). Cells were then collected, washed twice with PBS, and stained for live and dead cells with the LIVE/DEAD fixable green dead cell stain kit (Thermo Fisher Scientific) according to the manufacturer's instructions. Flow cytometry analysis was then performed on samples using the Sony ID7000 Full spectrum flow cytometer. Live cells were gated, and the median fluorescence intensity (MFI) of Cy3 was obtained.

Cy3-MTL-CEBPA uptake over time

Cell viability was determined with Trypan Blue staining (Gibco) and cells were seeded at a concentration of 5x10^5 cells/mL 16-24 hours prior to start of treatment. Cells were washed once in PBS and resuspend in serum-free RPMI-1640 with 1% ABAM. Cy3-MTL-CEBPA was added to culture media for a final concentration of 2 μg/mL and cells were incubated for 10 minutes, 30 minutes, 1 hour, 2 hours, 4 hours, 6 hours, or 24 hours. Cells were then collected, washed twice with PBS, and stained for live and dead cells with the LIVE/DEAD fixable green dead cell stain kit (Thermo Fisher Scientific) according to the manufacturer's instructions. Flow cytometry analysis was then performed on samples using the Sony ID7000 Full spectrum flow cytometer.

SUPPLEMENTAL FIGURES

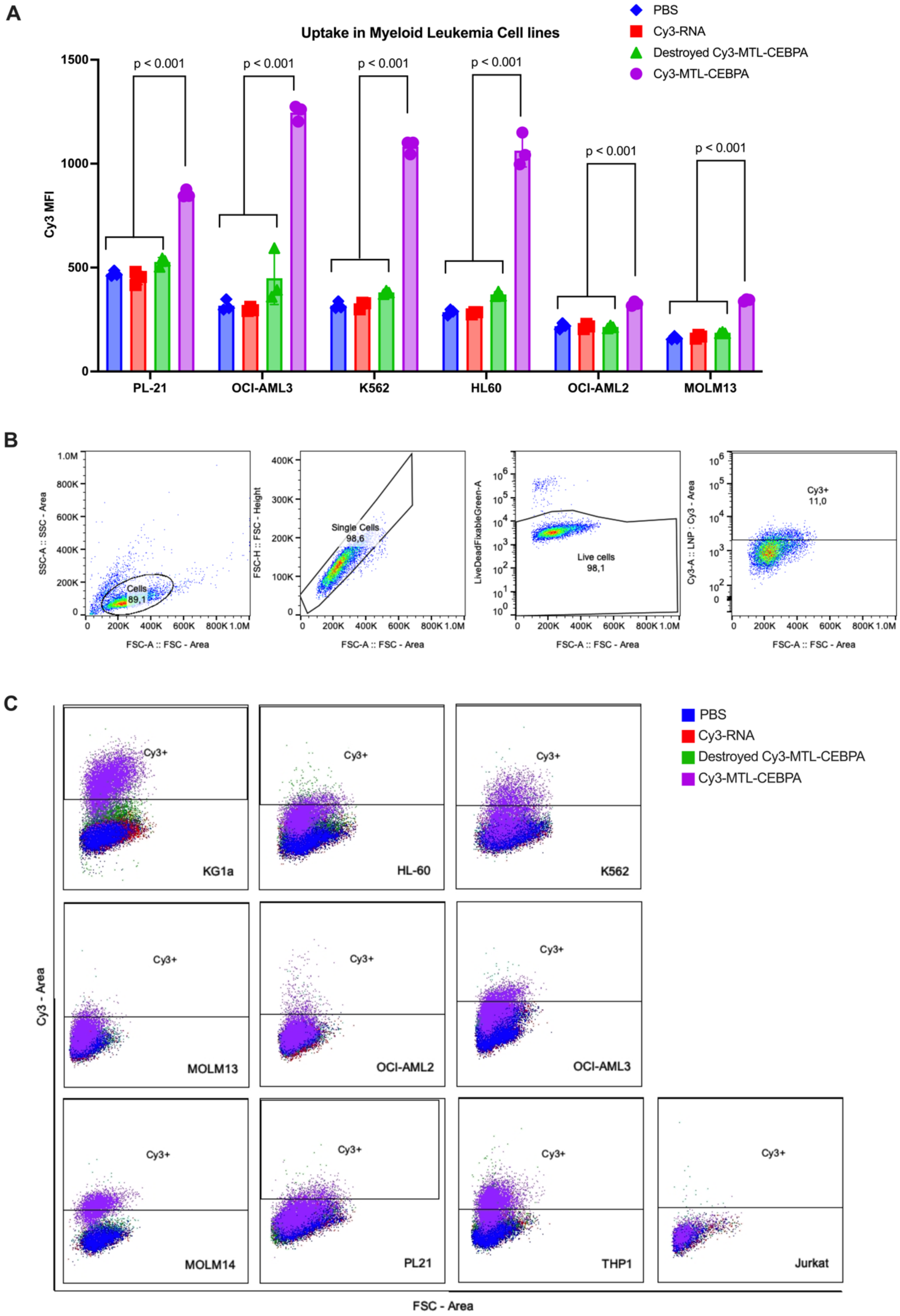

Figure S1. Uptake of Cy3-MTL-CEBPA in AML cell lines. (A) Delivery of fluorescently tagged CEBPA-51 saRNA in myeloid leukemia cell lines. Two-way ANOVA with Holm-Sidak Multiple Comparison Test; n=3. Data represented as mean ± SD. **(B)** Representative gating strategy for quantification of % of Cy3+ cells. **(C)** Representative dot plot maps of cell lines tested.

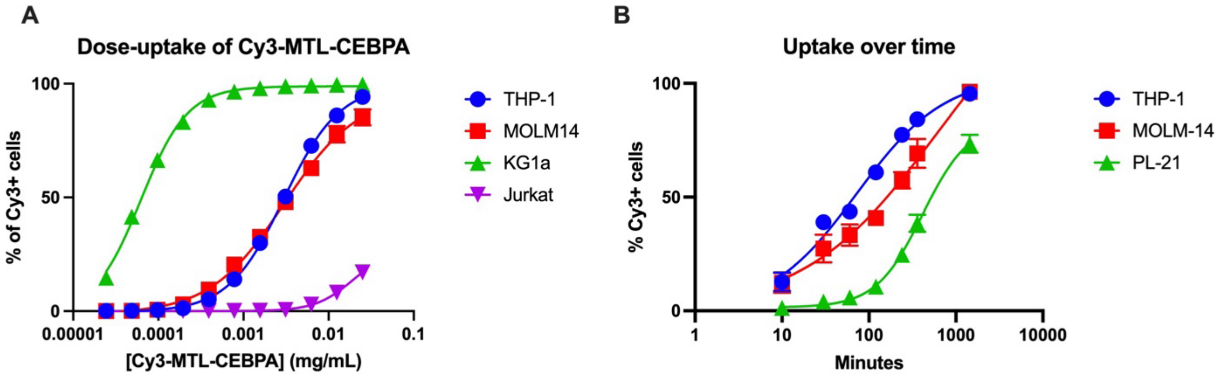

Figure S2. Cy3-MTL-CEBPA kinetics in AML cell lines. (A) Dose-response relationship of Cy3-MTL-CEBPA in THP-1, MOLM14, KG1a, and Jurkat cell lines. Data represented as mean ± SD. **(B)** Uptake of 2 μg/mL Cy3-MTL-CEBPA over time in THP-1, MOLM14, and PL21 cell lines. Data represented as mean ± SD.

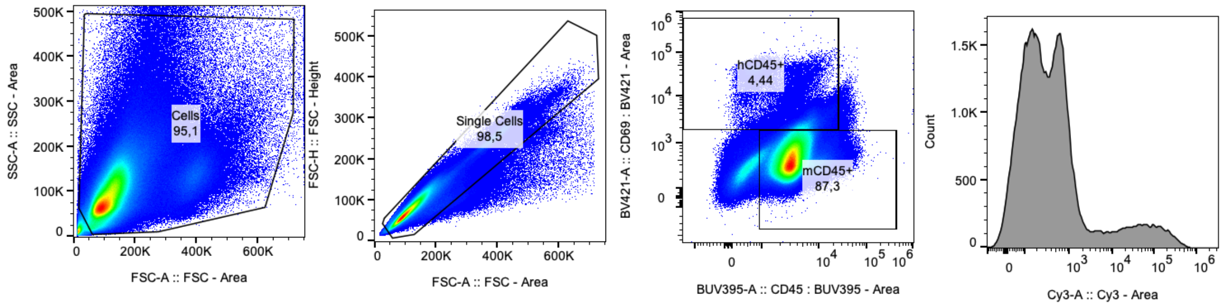

Figure S3. ***In vivo*** **uptake of Cy3-MTL-CEBPA in PDX mouse model.** Gating Strategy for quantification of Cy3 median fluorescence intensity (MFI) for Cy3-MTL-CEBPA uptake in PDX models.

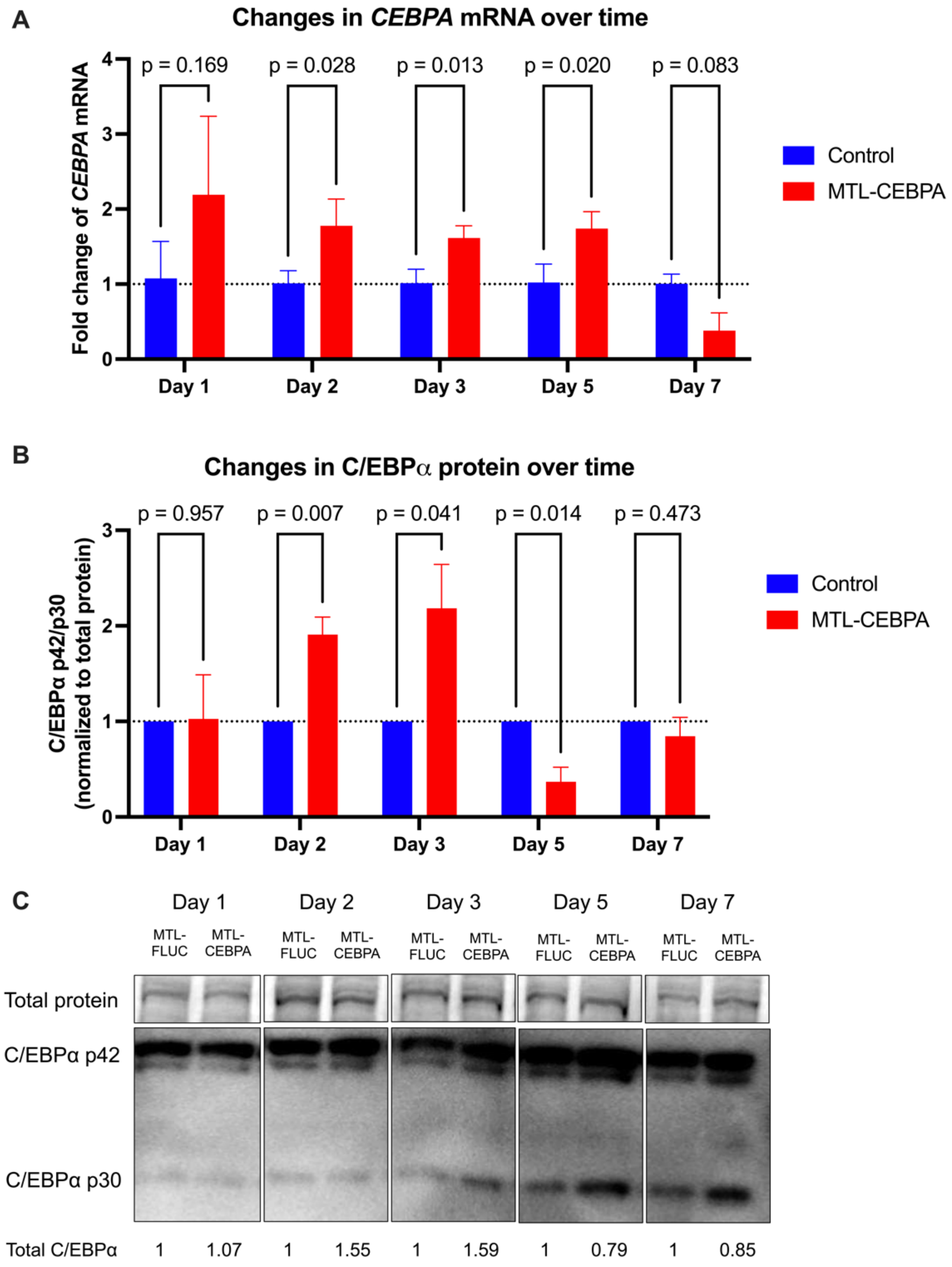

Figure S4. Changes in *CEBPA* expression over time. (A) *CEBPA* mRNA expression in MOLM-14 cells over time after a single treatment with 10 µg/mL MTL-CEBPA. Two-tailed unpaired student t-test; n=3. Data represented as mean ± SEM. **(B)** C/EBPα p42/p30 isoform ratio in MOLM-14 cells over time after a single treatment with 10 µg/mL MTL-CEBPA. Two-tailed unpaired student t-test; n=3. Data represented as mean ± SEM. **(C)** Representative western blot of C/EBPα over time. Relative total C/EBPα values written below blot.

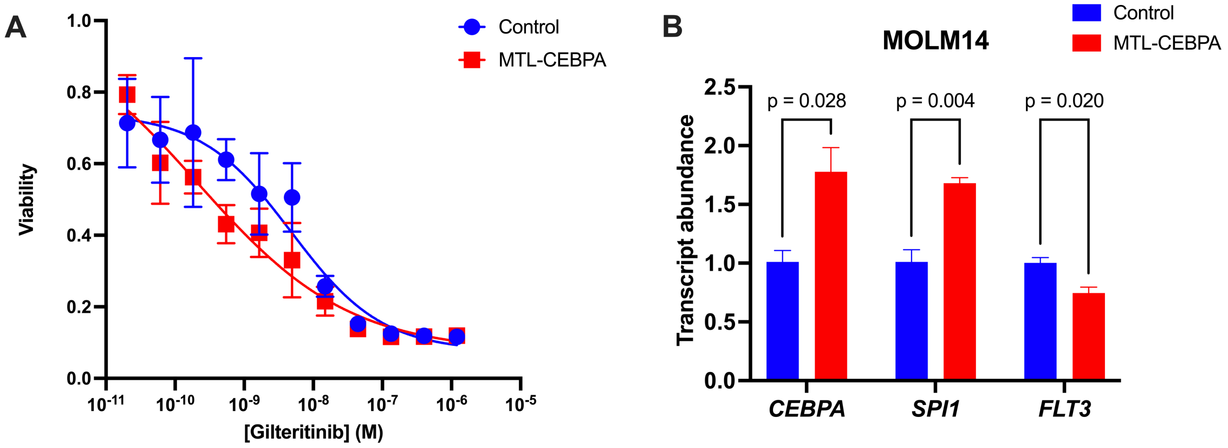

Figure S5. Therapeutic effect of MTL-CEBPA in MOLM14. (A) Gilteritinib dose-response relationship of MOLM14-venus cells treated with 2 µg/mL of MTL-CEBPA (blue) vs cells treated with equal concentration of control (MTL-FLUC). Data represented as mean ± SD. **(B)** Change in *CEBPA, SPI1,* and *FLT3* mRNA expression in MOLM-14 cells 72 hours after 10 µg/mL MTL-CEPBA treatment as compared to control (MTL-FLUC). Two-tailed unpaired student t-test; n=3. Data represented as mean ± SEM.

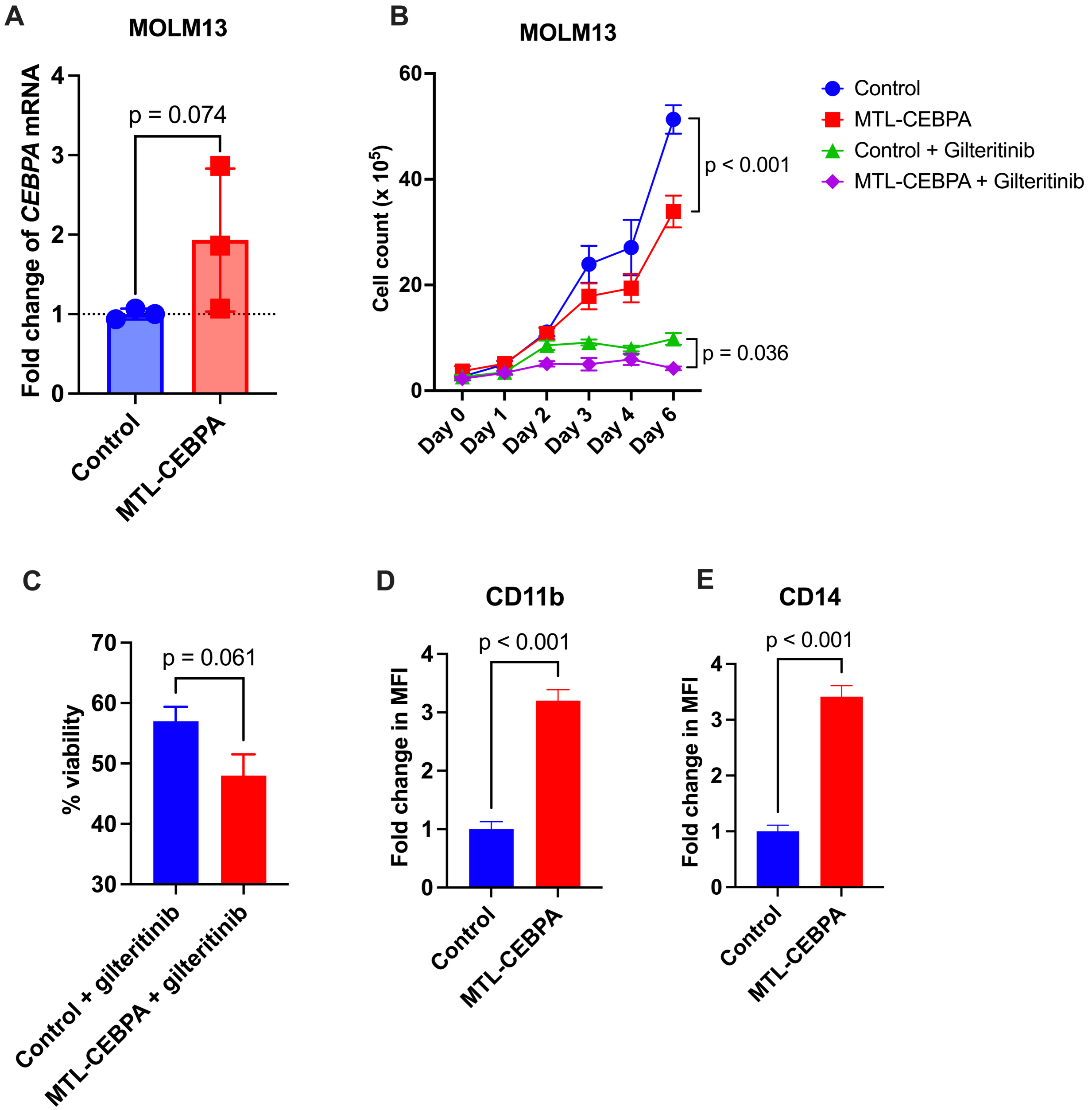

Figure S6. Therapeutic effect of MTL-CEBPA in MOLM-13. (A) MTL-CEBPA upregulates *CEBPA* mRNA, relative to cells treated with equal concentration (2 µg/mL) of control (MTL-FLUC) in MOLM13. Two-way unpaired student t-test; n=3. Data represented as mean ± SD. **(B)** Growth curves for MOLM-13 cells following treatment with 10 µg/mL of MTL-CEBPA or control (MTL-FLUC) with and without 5 nM gilteritinib. Two-way ANOVA with Holm-Sidak Multiple Comparison Test; n=6. Data represented as mean ± SEM. **(C)** Percent viability of cells on day 6. Two-way unpaired student t-test; n=6. Data represented as mean ± SEM. **(D)** Fold change in CD11b MFI and **(E)** CD14 MFI in MOLM-13 cells following treatment with 10 µg/mL MTL-CEBPA or control (untreated). Two-way unpaired student t-test; n=2-3. Data represented as mean ± SEM.

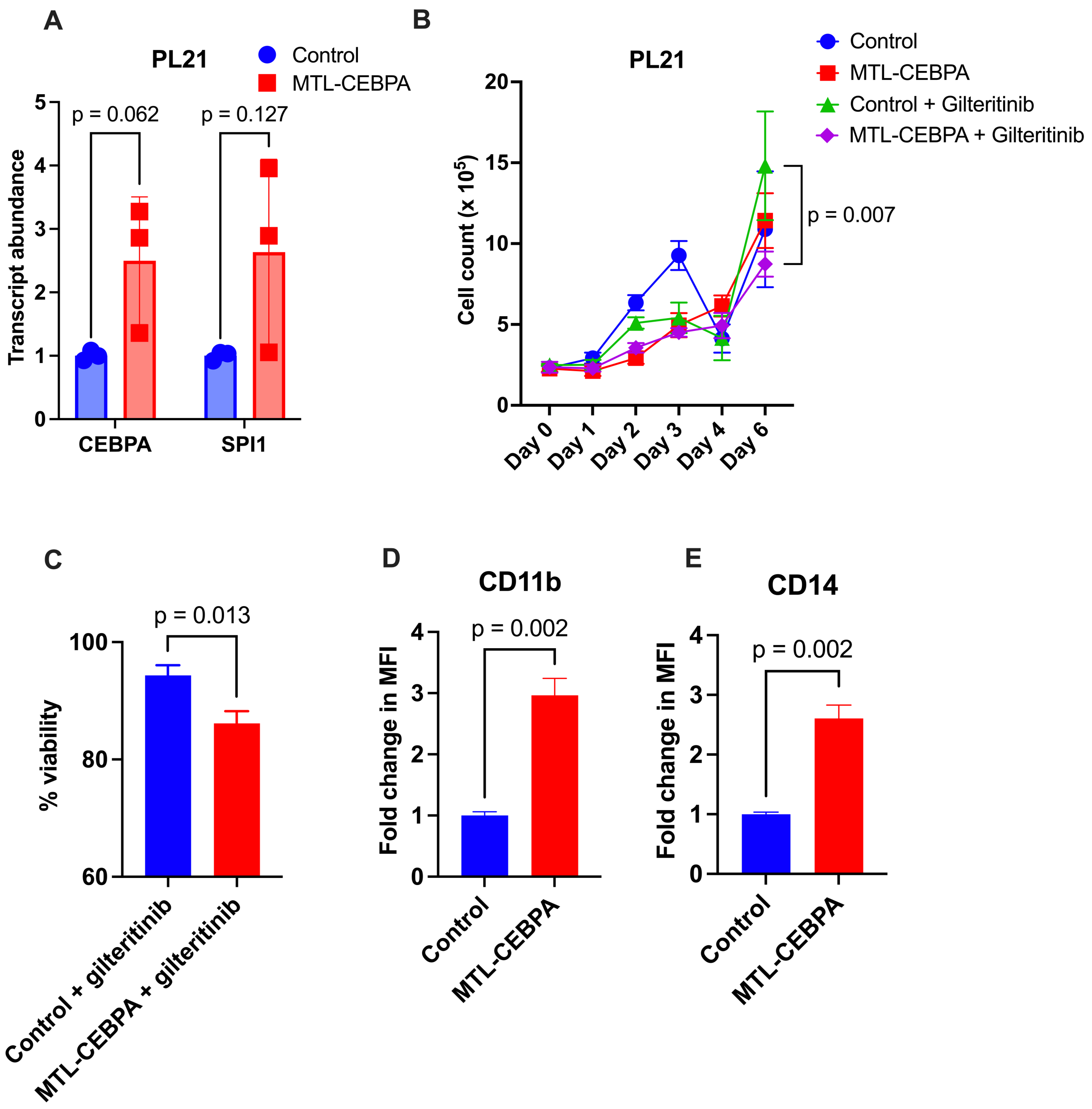

Figure S7. Therapeutic effect of MTL-CEBPA in PL-21. (A) MTL-CEBPA upregulates *CEBPA* and *SPI1* mRNA, relative to cells treated with equal concentration (2 µg/mL) of control (MTL-FLUC) in PL-21. Two-way unpaired student t-test; n=3. Data represented as mean ± SD. **(B)** Growth curves for PL-21 cells following treatment with 10 µg/mL of MTL-CEBPA or control (MTL-FLUC) with and without 5 nM gilteritinib. Two-way ANOVA with Holm-Sidak Multiple Comparison Test; n=6. Data represented as mean ± SEM. **(C)** Percent viability of cells on day 6. Two-way unpaired student t-test; n=6. Data represented as mean ± SEM. **(D)** Fold change in CD11b MFI and **(E)** CD14 MFI in PL-21 cells following treatment with 10 µg/mL MTL-CEBPA or control (untreated). Two-way unpaired student t-test; n=2-3. Data represented as mean ± SEM.

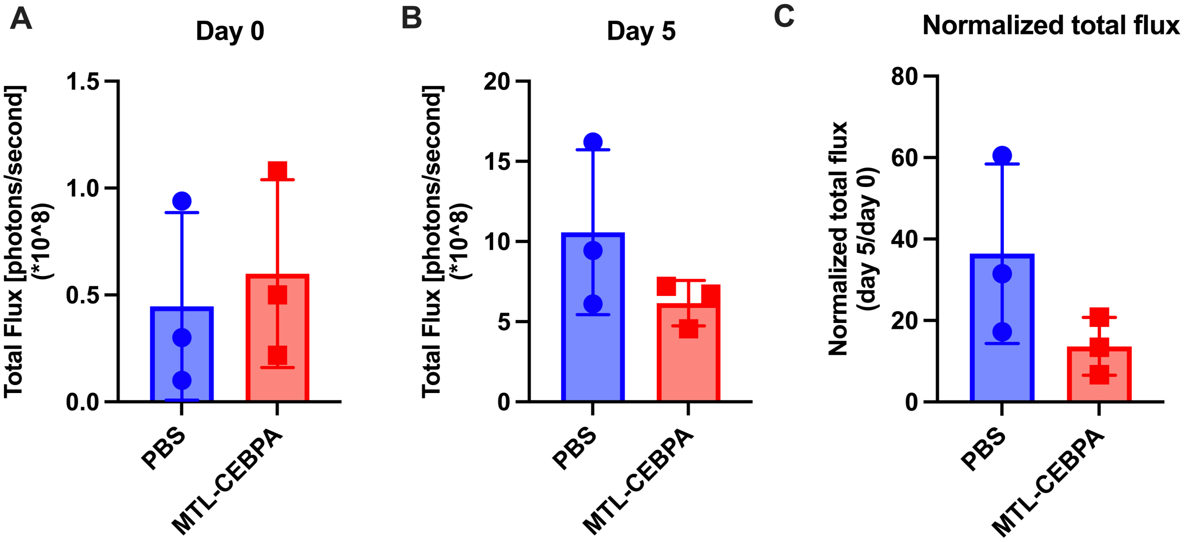

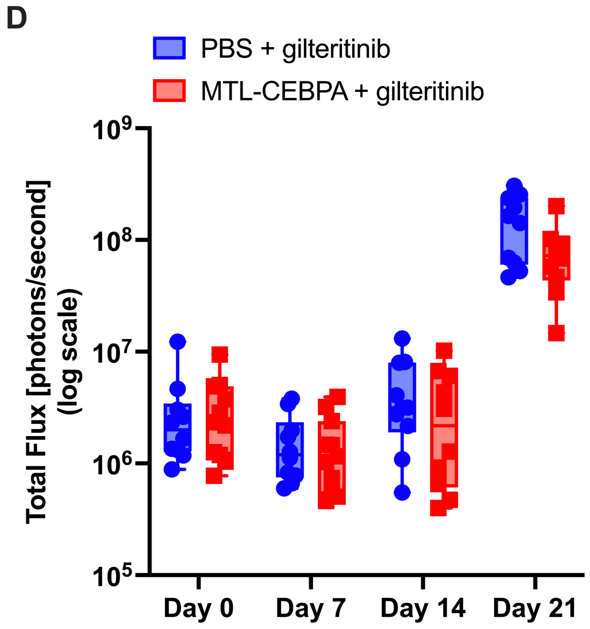

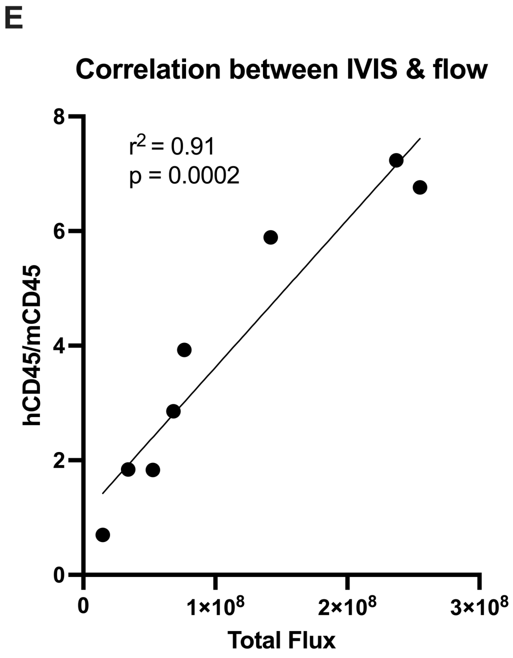

Figure S8. (A) Bioluminescence signal (total flux) measured on day 0 of MOLM-14-luc-engrafted NSG mice. **(B)** Total flux measured on day 5 of MOLM-14-luc-engrafted mice following intravenous administration of 3 mg/kg MTL-CEBPA or PBS every 2 days. **(C)** Normalized total flux for MOLM-14-luc-engrafted NSG mice. **(D)** Total flux values for results shown in Figure 5. **(E)** Correlation between total flux (obtained through in vivo imaging system (IVIS)) and ratio of human CD45 to mouse CD45 cells in the bone marrow of MOLM-14-luc-engrafted mice.

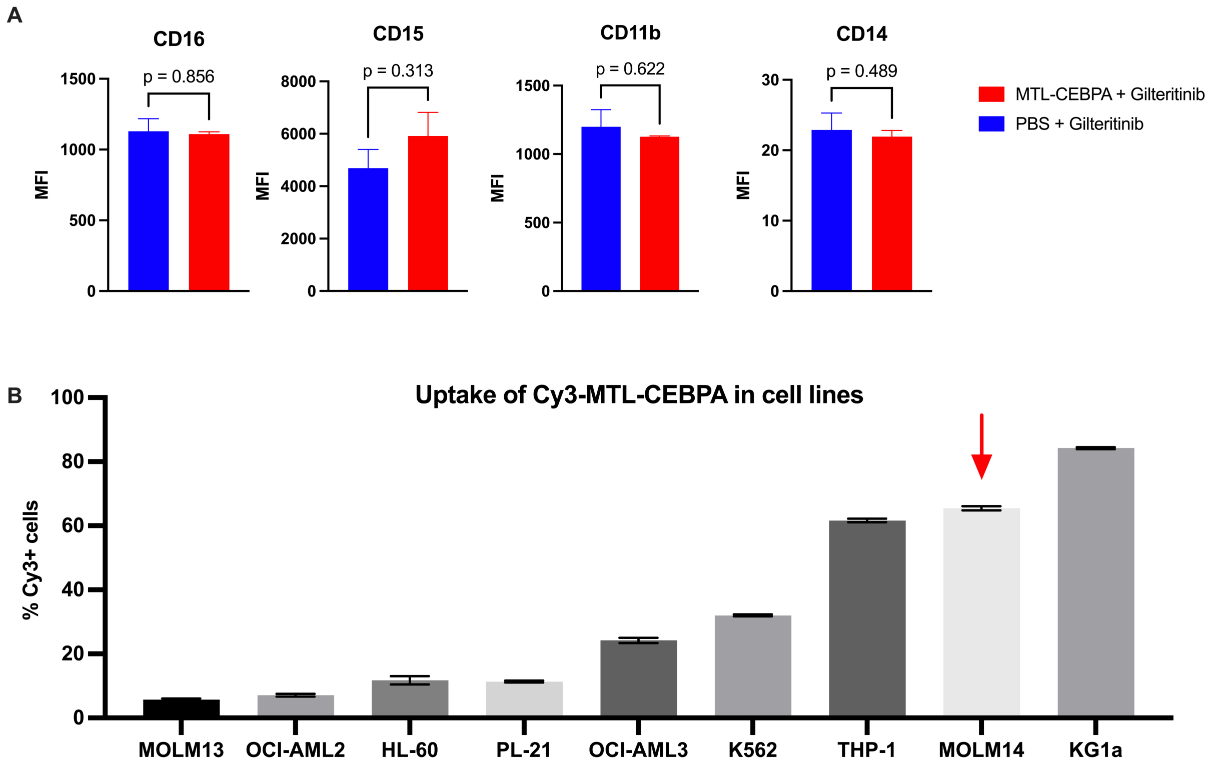

Figure S9. (A) Expression of myeloid-specific cell surface markers CD16, CD15, CD11b, CD14 in human MOLM-14-Ven-Luc cells isolated from the bone marrow of xenograft mice at day 21. **(B)** Percent uptake of Cy3-MTL-CEBPA in AML cell lines. Red arrow showing percent uptake for MOLM14 cell line.

SUPPLEMENTAL TABLES

Table S1. Summary table of FLT3 inhibitors used in study.

FLT3 inhibitor	Targets	Use in AML
gilteritinib	FLT3, AXL, ALK, and LTK[1]	Relapsed/refractor AML (ADMIRAL trial)[2]
midostaurin	FLT3, PKC, KDR, KIT, VEGFR, PDGFR[3]	Newly diagnosed AML (RATIFY trial)[4]
sorafenib	FLT3, RAF, RET, KIT, VEGFR, PDGFR[5]	Post-transplant maintenance (SORMAIN trial)[6]
quizartinib	FLT3, KIT, PDCFR[7]	Newly diagnosed AML (QuANTUM-First trial)[8]
ponatinib	FLT3, BCR-ABL, KIT, FGFR1, PDGFR[9]	Under evaluation (used in CML and Ph+ ALL)[10]
sunitinib	FLT3, PDGFR, VEGFR, KIT[11]	Under evaluation (currently used in solid tumours)
crenolanib	FLT3, PDGFR[12]	Under evaluation
lestaurtinib	FLT3, TRK, JAK2, PKC, KDR, PDGFR	Withdrawn from use[13]

SUPPLEMENTAL REFERENCES

1 Lee, L. Y., Hernandez, D., Rajkhowa, T., Smith, S. C., Raman, J. R., Nguyen, B., Small, D. & Levis, M. Preclinical studies of gilteritinib, a next-generation FLT3 inhibitor. *Blood* **129**, 257-260 (2017). https://doi.org/10.1182/blood-2016-10-745133

2 Perl, A. E., Martinelli, G., Cortes, J. E., Neubauer, A., Berman, E., Paolini, S., Montesinos, P., Baer, M. R., Larson, R. A., Ustun, C. *et al.* Gilteritinib or Chemotherapy for Relapsed or Refractory <i>FLT3</i>-Mutated AML. *New England Journal of Medicine* **381**, 1728-1740 (2019). https://doi.org/doi:10.1056/NEJMoa1902688

3 Schlenk, R. F. & Kayser, S. Midostaurin: A Multiple Tyrosine Kinases Inhibitor in Acute Myeloid Leukemia and Systemic Mastocytosis. *Recent Results Cancer Res* **212**, 199-214 (2018). https://doi.org/10.1007/978-3-319-91439-8_10

4 Stone, R. M., Mandrekar, S. J., Sanford, B. L., Laumann, K., Geyer, S., Bloomfield, C. D., Thiede, C., Prior, T. W., Döhner, K., Marcucci, G. *et al.* Midostaurin plus Chemotherapy for Acute Myeloid Leukemia with a <i>FLT3</i> Mutation. *New England Journal of Medicine* **377**, 454-464 (2017). https://doi.org/doi:10.1056/NEJMoa1614359

5 Zhu, Y.-j., Zheng, B., Wang, H.-y. & Chen, L. New knowledge of the mechanisms of sorafenib resistance in liver cancer. *Acta Pharmacologica Sinica* **38**, 614-622 (2017). https://doi.org/10.1038/aps.2017.5

6 Burchert, A., Bug, G., Fritz, L. V., Finke, J., Stelljes, M., Röllig, C., Wollmer, E., Wäsch, R., Bornhäuser, M., Berg, T. *et al.* Sorafenib Maintenance After Allogeneic Hematopoietic Stem Cell Transplantation for Acute Myeloid Leukemia With <i>FLT3</i>–Internal Tandem Duplication Mutation (SORMAIN). *Journal of Clinical Oncology* **38**, 2993-3002 (2020). https://doi.org/10.1200/jco.19.03345

7 Kampa-Schittenhelm, K. M., Heinrich, M. C., Akmut, F., Döhner, H., Döhner, K. & Schittenhelm, M. M. Quizartinib (AC220) is a potent second generation class III tyrosine kinase inhibitor that displays a distinct inhibition profile against mutant-FLT3, -PDGFRA and -KIT isoforms. *Molecular Cancer* **12**, 19 (2013). https://doi.org/10.1186/1476-4598-12-19

8 Erba, H. P., Montesinos, P., Kim, H.-J., Patkowska, E., Vrhovac, R., Žák, P., Wang, P.-N., Mitov, T., Hanyok, J., Kamel, Y. M. *et al.* Quizartinib plus chemotherapy in newly diagnosed patients with FLT3-internal-tandem-duplication-positive acute myeloid leukaemia (QuANTUM-First): a randomised, double-blind, placebo-controlled, phase 3 trial. *The Lancet* **401**, 1571-1583 (2023). https://doi.org/10.1016/S0140-6736(23)00464-6

9 Gao, Y., Ding, Y., Tai, X.-r., Zhang, C. & Wang, D. Ponatinib: An update on its drug targets, therapeutic potential and safety. *Biochimica et Biophysica Acta (BBA) - Reviews on Cancer* **1878**, 188949 (2023). https://doi.org/https://doi.org/10.1016/j.bbcan.2023.188949

10 Chevallier, P., Thiebaut, A., François, S., Chantepie, S., Rubio, M. T., Labussiere-Wallet, H., Brissot, E., Maillard, N., Huynh, A., Coiteux, V. *et al.* A phase II study of ponatinib for prevention of relapse after allotransplantation in FLT3 internal tandem duplication mutation positive (FLT3-ITD+) acute myeloid leukemia: the

PONALLO trial. *Haematologica* (2025). https://doi.org/10.3324/haematol.2025.287681

11 Yu, J., Jiang, P. Y. Z., Sun, H., Zhang, X., Jiang, Z., Li, Y. & Song, Y. Advances in targeted therapy for acute myeloid leukemia. *Biomarker Research* **8**, 17 (2020). https://doi.org/10.1186/s40364-020-00196-2

12 Galanis, A., Ma, H., Rajkhowa, T., Ramachandran, A., Small, D., Cortes, J. & Levis, M. Crenolanib is a potent inhibitor of FLT3 with activity against resistance-conferring point mutants. *Blood* **123**, 94-100 (2014). https://doi.org/10.1182/blood-2013-10-529313

13 Levis, M., Ravandi, F., Wang, E. S., Baer, M. R., Perl, A., Coutre, S., Erba, H., Stuart, R. K., Baccarani, M. & Cripe, L. D. Results from a randomized trial of salvage chemotherapy followed by lestaurtinib for FLT3 mutant AML patients in first relapse. *Blood* **114**, 788 (2009).
